# Effect of Alfalfa Hay and Starter Feeding Intervention on Gastrointestinal Microbial Community, Growth and Immune Performance of Yak Calves

**DOI:** 10.3389/fmicb.2020.00994

**Published:** 2020-06-04

**Authors:** Zhanhong Cui, Shengru Wu, Jilan Li, Qi-En Yang, Shatuo Chai, Lei Wang, Xun Wang, Xiaowei Zhang, Shujie Liu, Junhu Yao

**Affiliations:** ^1^Qinghai Academy of Animal Science and Veterinary Medicine, Qinghai University, Xining, China; ^2^College of Animal Science and Technology, Northwest A&F University, Yangling, China; ^3^Northwest Plateau Institute of Biology, Chinese Academy of Sciences, Xining, China

**Keywords:** yak calves, alfalfa, starter feeding, gastrointestinal microbiota, immune homeostasis, growth performance

## Abstract

The present study aims to evaluate the effects of different early weaning paradigms, which supplied with extra alfalfa hay, or starter feeding, or both alfalfa hay and starter feeding, along with the milk replacer, on the gastrointestinal microbial community, growth, and immune performance of yak calves. Twenty 30-day-old male yak calves were randomly assigned to four groups, including the control (CON), alfalfa hay (A), starter feeding (S), and starter plus alfalfa hay (SA) groups. The gastrointestinal microbial colonization, the gastrointestinal development and function, and the growth and immune performance of all the yak calves were separately measured. Supplementation with alfalfa and starter feeding during the pre-weaning period significantly increased body weight, body height, body length, and chest girth. The significantly improved rumen fermentation and promoted intestinal digestion-absorption function in alfalfa and starter feeding groups, including the identified significantly increased concentrations of ruminal total volatile fatty acid (VFA); the significantly increased concentrations and proportions of acetate, butyrate, and isovalerate; the increased α-amylase activities in the duodenum, jejunum, and ileum; the increased papillae length and width of rumen epithelium and rumen wall thickness; and the increased villus height and crypt depth of the duodenum, jejunum, and ileum, could all contribute to promote the growth of calves. These significant improvements on rumen fermentation and intestinal digestion-absorption function could be further attributed to the increased proliferation of starch-decomposing, and cellulose- or hemicellulose-decomposing bacteria identified in the rumen, jejunum, and ileum. Furthermore, based on the expression of intestinal inflammatory cytokines and the rumen epithelial RNA sequencing results, alfalfa supplementation reduced the occurrence of ruminal and intestinal inflammation, whereas starter feeding supplementation was mainly beneficial to the differentiation of immune cells and the improved immune function. Meanwhile, the significantly altered relative abundances of genera in the SA group, including increased relative abundance of *Limnobacter*, *Escherichia*/*Shigella*, and *Aquabacterium* in the rumen and increased relative abundance of *Coprococcus, Pseudobutyrivibrio*, *Flavonifractor*, *Synergistes*, and *Sutterella* in jejunum, were able to reduce gastrointestinal inflammation and enhance the immune function, which enhanced the immune function of the yak calves fed with alfalfa and starter feeding. Overall, milk replacer supplemented with alfalfa and starter feeding during the pre-weaning period could alter gastrointestinal microbiota and then benefit the gastrointestinal development, digestion-absorption function, growth, and immune performance of the yak calves.

## Introduction

The yak, which is the major indigenous ruminant on the Qinghai-Tibetan Plateau in China, has been grazed and used by local herdsmen for meat, milk, and fuel for several centuries (Xue et al., 2005). Calves are important for the sustainable development of the yak industry. The quality of calf cultivation directly determines the performance of adults. However, the pre-weaning period for yak calves mainly occurs during maternal grazing and nursing, which lasts for 120–180 days on the Qinghai-Tibetan Plateau and is not beneficial to the estrus and mating of female yaks or the survival and growth of the calves. Adequate nutrition supplementation in the calves’ early life can have a long-term impact on the dairy calves. For instance, the gastrointestinal development, the functional transition from metabolizing milk glucose to metabolizing short-chain fatty acids, growth, and the immunity of the calves could all be affected by the intake of starter feeding and alfalfa hay during the pre- and post-weaning phases (3–70 days after birth) (Kargar and Kanani, 2019). Considering the nutrition deficiency of the yak calf by long-term (120–180 days) maternal grazing and nursing on Qinghai-Tibetan Plateau and the growing demand to improve the growth performance of yaks, early weaning with mixed rations of available roughage or grains in barn feeding paradigms, whose effects on the growth and health of lambs and dairy calves have been widely suggested (Khan et al., 2016), was presented as an alternative to natural grazing and was supposed to be beneficial to the growth and carcass characteristics of yaks.

Individually supplying alfalfa hay or starter feeding to the ruminants during the pre-weaning period is controversial, due to their differential roles in promoting rumen development (Norouzian et al., 2011) and maintaining gastrointestinal immune homeostasis (Liu et al., 2017) in lambs and dairy calves during the pre- and post-weaning phases. Briefly, it has been noted that alfalfa hay promotes epithelium and muscular development of the rumen in dairy calves. However, alfalfa hay digestion by microorganisms does not provide sufficient concentrations of ruminal volatile fatty acids (VFAs), especially butyrate, required for optimal papillae development in dairy calves until 2 weeks later after weaning (Jahani-Moghadam et al., 2015; Mirzaei et al., 2015; Hosseini et al., 2016). The starter feeding provides readily fermentable carbohydrates for the production of VFAs, especially butyrate, which is necessary to stimulate papillae development. However, ruminants that are only fed starter feeding are at a greater risk of developing ruminal or metabolic acidosis and parakeratosis, which may severely compromise gastrointestinal functions and lead to harmful effects on the development and health of the pre- and post-weaning lambs from 10 to 56 days (Liu et al., 2017; Wang et al., 2017). Overall, considering their potentially unique and complementary effects on rumen development, gastrointestinal immune homeostasis, and the microbial colonization of calves, the present research focuses on the most suitable weaning feeding paradigm for yak calves, which were fed with extra alfalfa hay, or starter feeding, or both of alfalfa hay and starter feeding along with the milk replacer during their early lives.

Alfalfa hay and starter feeding supplementation during the pre-weaning period have a crucial and long-term impact on various biological functions (Saro et al., 2018), which could result from the altered gastrointestinal microbiota and enhanced rumen fermentation or intestinal nutrients utilization during early life. In dairy calves during the pre-weaning (7 weeks of age), weaning transition (8 weeks of age), and post-weaning (9–11 weeks of age) phases, the supplementation of alfalfa hay and other fiber carbohydrates could increase the ruminal abundance of *Bacteroidetes*, which is an important cellulose-decomposing bacteria, and further increase the rumen pH and contribute to rumen health and rumen epithelium development (Kim et al., 2016). Meanwhile, in dairy calves during the pre-weaning phase (7, 28, 49, and 63 days), supplementation with starter feeding could increase *Megasphaera*, *Sharpea*, and *Succinivribrio*, which respond by utilizing the rumen fermentable carbohydrates (Dias et al., 2017), and further enhance the rumen fermentation and promote growth of ruminants. Considering that the characteristic ruminal and intestinal microbiota of yak result from a history of long-term grazing (Long et al., 2008; Xue et al., 2017; Huang and Li, 2018), barn feeding with alfalfa hay, starter feeding, and milk replacer has the potential to influence the gut microbiota and further contribute to tremendous gastrointestinal functional alterations, which have rarely been studied in the yak calves.

Along with the supplementation of milk replacer, by supplying with extra alfalfa hay, or starter feeding, or alfalfa hay and starter feeding, the present study aims (1) to compare the effects of different early weaning paradigms on gastrointestinal development, nutrient utilization, and immune homeostasis in yak calves and screen out the most suitable weaning feeding paradigm for yak calves, (2) to reveal the roles of the altered gastrointestinal microbiota in regulating the gastrointestinal function, nutrient utilization, immune homeostasis, and development of pre-weaning yaks that reflect the individual or simultaneous supplementation with alfalfa and starter feeding.

## Materials and Methods

### Ethics Approval Statement

This study was carried out in accordance with the recommendations of the Administration of Affairs Concerning Experimental Animals (Ministry of Science and Technology, China, revised 2004). The protocol was approved by the Institutional Animal Care and Use Committee of the Northwest A&F University (protocol number NWAFAC1118).

### Animals and Experimental Design

Before the commencement of the trial, all the yak calves were fed with the maternal milk by maternal nursing only at the Datong Yak Breeding Farm of Qinghai Province. Meanwhile, we have performed a power analysis to justify the sample size by using the pwr.anova.test [*k* = 4, *n* = 5, *f* = 1 (large effect sizes), sig.level = 0.05] in the pwr package according to Cohen (1988), where *k* is the number of groups and *n* is the common sample size in each group. The results indicated that the power is 0.927, which indicated that *n* = 5 is enough to obtain the credible result. Herein, 20 male yak calves at the age of 30 days [Body weight (BW) of 34.86 ± 2.06 kg, mean ± standard deviation (SD)] were then randomly selected and assigned to four groups, with five calves per group. The five calves in each group were individually feeding in five different pens. The control group was supplied with the milk replacer; the alfalfa (A) group was supplied with milk replacer and alfalfa; the starter feeding (S) group was supplied with milk replacer and starter; and the starter plus alfalfa (SA) group was supplied with milk replacer, starter, and alfalfa hay. The yak calves in each group were fed twice a day at 08:00 h and 16:30 h. All yak calves in the four treatments, in addition to their respective access to the starter feed and/or alfalfa hay, were supplied twice a day at 08:00 and 16:30 with milk replacer constituted from 100 to 350 g milk replacer powder (the supplementation of milk replacer were increased along with the increasing body weight) dissolved in 1 L 60°C water. Water was supplied *ad libitum* to the yak calves during the experimental period. The experiment was performed from June to October and lasted for 90 days. At the end of the experiment, all yak calves were weighed, and the body size indexes, including the body height, body length, and chest girth, were recorded. Meanwhile, during the experimental period, the feed offered was recorded daily, and the residue was collected daily, pooled, and weighed at 3-day intervals for the calculation of the averaged daily feed intake over the 3 days. This approach resulted in a total of 30 measures of the feed intakes for each of the calves over the whole period, and the mean of those 30 intakes was used an individual replicate for the statistical analysis on the difference of feed intake between four treatments.

Composites of the starter feed, alfalfa hay, and milk replacer were measured (AOAC International, 2000) for dry matter (oven method 930.15), sugar (colorimetric method), crude protein (Kjeldahl method 988.05), ether extract (alkaline treatment with Röse-Gottlieb method 932.06 for MR; diethyl ether extraction method 2003.05 for starter and alfalfa hay), NDF with ash without sodium sulfite or α-amylase, ADF with ash, starch (α-amylase method), Calcium (Ca) and Phosphorus (P) (dry ashing, acid digestion, and analysis by inductively coupled plasma, method 985.01), and the details of the nutrient composition were given in Table 1.

**TABLE 1:** Nutrient composition of the alfalfa, starter, and milk replacer used in the present study.

Items (% of dry matter)	Milk replacer^a^	Alfalfa hay	Starter feed
Dry matter (% as-fed)	94.00	93.80	87.90
Sugar	–	–	6.50
Starch	–	–	40.50
Crude protein	24.00	12.50	20.00
Ether extract	16.00	0.90	4.70
Neutral detergent fiber	–	56.45	10.90
Acid detergent fiber	–	40.40	4.10
Calcium	0.60∼3.00	0.98	0.80
Phosphorus	0.50∼2.00	0.18	0.45
Lysine	2.20	0.85	1.06
Methionine	1.00	0.17	0.33

*^*a*^The milk replacer was stored in powder, and was consisted with the whole milk powder, whey powder, protein concentrate, vitamin A (VA), VD3, VE, nicotinic acid, pantothenic acid, lysine, methionine, threonine, sodium chloride, copper, zinc manganese, and iron.*

### Sample Collection

After the calves had been fed for 90 days, the jugular venous blood samples of all yak calves were collected before morning feeding, and the plasma samples were prepared. Briefly, the blood samples were collected into 5 mL vacutainer tubes with the chelating agent EDTA-K2, and then the samples were centrifuged at 3500 × *g* for 15 min at 4°C for plasma collection. Then the calves were weighted, euthanized by exsanguination after intravenous administration of 10% chloral hydrate solution (100 mg chloral hydrate/kg body weight; Sigma, United States), and immediately dissected.

First, the 30 cm^2^ ruminal epithelial tissue samples at the same position were also collected for determination of ruminal morphology (Ishii et al., 2005). Then, following removal of the intestinal contents to prevent contamination, the middle complete duodenal, jejunal, and ileal segments were collected in lengths of 3 cm for further intestinal histological processing. The above ruminal epithelial tissue samples and intestinal segments were all fixed in 10% buffered formalin for at least 48 h before the determination of rumen and intestinal morphology. Specifically, to ensure that the sampling sites of intestines were consistent, we segmented the intestine into duodenum, jejunum, and ileum as following criterions: the duodenum tissue was the first 15 cm of the small intestine beginning at the pyloric sphincter; the ileum tissue was the distal 15 cm portion of the small intestine that ended at the ileocecocolic junction. About 15 cm from the middle of the small intestine was sampled as the jejunum tissue. Then the middle complete intestinal segments in lengths of 3 cm were separately collected for intestinal morphology.

Second, the residual 12 cm sampled duodenal, jejunal, and ileal segments were used to collect duodenal, jejunal, and ileal mucosal samples. The duodenal, jejunal, and ileal mucosal samples and the rumen dorsal epithelial tissue samples at the same position were used for further RNA sequencing and enzyme-linked immunosorbent assay (ELISA) tests. Briefly, the duodenal, jejunal, and ileal sections were cut along the dorsal line, and the contents were emptied. The fundic region of these tissues was washed with ice-cold 0.9% saline, and the mucosal was scraped using a sterile glass slide. Specifically, in order to avoid the potential influence of Payer’s patch on the subsequent measurement, the presence of Payer’s patch was checked first, and only the patch-free segment of jejunum was used for sample collection and further analysis.

Third, rumen fluid was collected and strained through 4 layers of sterile cheesecloth. The pH of the rumen fluid was measured immediately with a mobile pH meter (HI 9024C; HANNA Instruments, Woonsocket, RI, United States); meanwhile, another 5 mL of rumen fluid was collected for VFAs and NH_3_-N analyses. Specifically, a solute with metaphosphoric acid and crotonic acid was added to 2 mL of these 5 mL rumen fluid samples before further analyses of the VFA concentrations.

At last, the rumen fluid, abomasumal content, duodenal content, jejunal content, and ileal content samples were collected for digestive enzyme analyses. Meanwhile, more tubes of rumen fluid, jejunal content, and ileal content samples were collected for DNA extraction and further 16S rRNA gene sequencing.

The duodenal, jejunal, and ileal segments and the rumen dorsal epithelial tissue samples were fixed in 10% buffered formalin and stored at 4°C until the analysis of ruminal and intestinal morphology. Besides, all the other collected samples were first stored in liquid nitrogen for 24 h and then stored in −80°C until analyses.

### Measurement of Blood and Plasma Biochemical and Immune Indexes

The glutamic-pyruvic transaminase (ALT), glutamic oxalacetic transaminase (AST), total protein (TP), albumin (ALB), globulin (GLO), lactic dehydrogenase (LDH), and alkaline phosphatase (ALP) contents in the blood were determined using an automatic biochemical analyzer (Cobas 601, Roche, Germany) with the help of Qinghai Provincial People’s Hospital. Meanwhile, the immune globulin G (IgG), immune globulin A (IgA), immune globulin M (IgM), nitric oxide (NO), and lysozyme contents were measured by spectrophotometric methods (Jiancheng Biological Engineering Research Institute, Nanjing, China). The tumor necrosis factor α (TNFα), interleukin 1β (IL-1β), IL-2, IL-4, alexin C3, and cortisol contents were measured by ELISA (Cloud-Clone Corporation, Houston, TX, United States).

### Determination of Rumen and Intestinal Morphology

After being fixed in 10% buffered formalin, the duodenal, jejunal, and ileal segments and the rumen dorsal epithelial tissue samples were dehydrated and cleared. Then, the rumen and intestinal samples were cut and inserted into cassettes, which were embedded in liquid paraffin. Next, 5 μm paraffin sections were cut using the microtome and stained with hematoxylin-eosin. The papillae length and width of the rumen epithelium, the thickness of the rumen base, and the height and crypt depth of the intestinal villus were determined using a phase contrast microscope (Nikon NiE200, Japan) (Wu et al., 2018).

### Determination of VFAs in Rumen Fluid

For the VFA and NH_3_-N measurements, the rumen fluid was centrifuged at 13,000 × *g* for 10 min. The VFAs were analyzed by an Agilent 6850 gas chromatograph (Agilent Technologies Inc., Santa Clara, CA, United States) equipped with a polar capillary column (HP-FFAP, 30 m × 0.25 mm × 0.25 μm) and a flame ionization detector (FID), as previously described (Xue et al., 2017). The NH_3_-N in the supernatant was quantified using a continuous-flow analyzer (SKALAR San, Skalar Co., Netherlands).

### Measurement of the Activities of Gastrointestinal Digestive Enzymes and Immune Cytokines

The activities of the gastrointestinal digestive enzymes, including the carboxymethyl cellulase, xylanase, and pectinase in the rumen; the pepsase and chymosin in the abomasum; and the α-amylase, trypsin, and lipase in the duodenum, jejunum, and ileum, were measured by spectrophotometric methods according to the manufacturer’s instructions (Jiancheng Biological Engineering Research Institute, Nanjing, China). Moreover, the sIgA, IL-2, IL-4, IL-10, TNF-α, and IFN-γ contents of the mucous membrane samples of the duodenum, jejunum, and ileum were measured by the microplate reader (Varioskan Flash, Thermo, United States) using ELISA kits (YuanMu Biological Technology Co. Ltd., Shanghai, China).

### Microbial DNA Extraction, 16S rRNA Gene Amplification of the V3 + V4 Region, Sequencing, and Bioinformatics Analysis

The rumen fluid, jejunal content, and ileal content samples of yak calves from four different treatments were used for DNA extraction using QIAamp DNA Stool Mini Kit (Qiagen, Germany). The integrity of the DNA was assessed using 1% agarosegel electrophoresis and the purity was assessed from the 260:280 nm ration (>1.8) using a NanoDrop ND2000 spectrophotometer (Thermo Scientific, United States). Furthermore, the concentration and amount of DNA samples were further detected by using Qbit fluorometer (Life Technologies, Mulgrave, VIC, Australia), and the DNA was stored at −80°C until it was used in sequencing analysis. Only the DNA samples with an optical density ratio at 260/280 nm > 1.8, with a concentration of 20 ng/μL (total volume was greater than 20 μL), and with ideal integrity were used in further analyses.

The amplicon library was prepared by polymerase chain reaction amplification of the V3–V4 region of the 16S rRNA gene using the primer set 341F (5′-CCTAYGGGRBGCASCAG-3′) and 806R (5′-GGACTACNNGGGTATCTAAT-3′) with barcodes (Yu et al., 2005; Sundberg et al., 2013). Briefly, considering that a large amount of archaeal such as methanogens were also colonized and play important roles in the rumen and gut of ruminant (Yu et al., 2005; Sundberg et al., 2013), the primer set 341F and 806R was selected to meanwhile obtain the information from the bacteria and archaeal. PCR reactions were performed in triplicate 20 μL mixture containing 4 μL of 5 × FastPfu Buffer, 2 μL of 2.5 mM dNTPs, 0.8 μL of each primer (5 μM), 0.4 μL of FastPfu Polymerase, and 10 ng of template DNA. Amplicons were extracted and purified from 2% agarose gels using the AxyPrep DNA Gel Extraction Kit (Axygen Biosciences, United States) and were quantified using QuantiFluor^TM^-ST (Promega, United States).

The 16S rRNA gene amplicons were used to determine the diversity of and to perform structural comparisons of the bacterial species present in each of the samples using a paired-end sequence (2 × 250) on an Illumina HiSeq 2500 platform according to the standard protocols. The sequence data was deposited and is available in the Sequence Read Archive (SRA) of NCBI with the accession project numbers PRJNA543073.

Raw fastq files were demultiplexed using the barcode sequence with the exact barcode matching parameter. The quality-filtering of raw tags was performed using Trimmomatic (version 0.36) (Bolger et al., 2014) with the following criteria: (i) bases off the start and end of a read below a threshold quality (Score < 2) were removed. (ii) The reads were truncated at any site receiving an average quality score < 2 over a 4 bp sliding window, discarding the truncated reads that were shorter than 100 bp.

Paired-end reads were merged using USEARCH (version 9.2.64)^1^ (Edgar and Flyvbjerg, 2015) with the default parameters. The primer sequences were identified and removed from the merged reads by using the subcommand “search_pcr” of USEARCH. Then reads that could not be merged were discarded, and the merged reads with more than two nucleotide mismatches in primer matching were removed.

These sequences were classified into operational taxonomic units (OTUs) at an identity threshold of a 97% similarity using the UPARSE software (Edgar, 2013). For each OTU, a representative sequence was screened and used to assign taxonomic composition by comparison with the RDP 16S Training set (v16) and the core set using the SINTA (Usearch V9.2.64) and PyNAST programmed algorithms (Caporaso et al., 2010; Edgar, 2016). Specially, we performed the “otutab rare” program by using USEARCH to keep the uniformization of data from different groups before these analyses. Subsequent analysis of alpha and beta diversity was performed based on the output of this normalized data by separately using USEARCH alpha_div (Edgar, 2010) and UniFrac metrics (Lozupone and Knight, 2005) in QIIME (version 1.9.1) (Caporaso et al., 2010). The relative abundance of different taxon for each sample was determined according to phylum, class, order, family, and genus. The microbiota was compared for beta diversity using the distance matrices generated from weighted UniFrac analysis and principal coordinated analysis (PCoA) (Lozupone et al., 2013). Linear discriminant analysis (LDA) and effect size (LEfSe) analysis (Paulson et al., 2013) was performed to estimate the effect size of species that contributed to the differences between the samples. The threshold of the LDA score was set at a default value of 2.0. The correlation between the identified genera and significantly altered performance of calves were analyzed by using spearman analysis.

### Rumen Epithelial RNA Isolation, Sequencing, and Bioinformatics Analysis

By using the Scotty website^2^ (Busby et al., 2013), the power analysis for the RNAseq experiment was also performed to identify the reliability of the RNA sequence by using five replications for each group in the present study. In brief, with expectation to detect more than 50% of accurate differentially expressed genes, five replicates sequenced to a depth of 40 million reads aligned to genes per replicate could obtain the most powerful results. These results indicated that five replicates sequenced to a depth of 40 million reads aligned to genes per replicate in the present study is enough to obtain the credible result of RNA sequencing as well. Total RNA from the rumen epithelial tissue samples of 20 yak calves (five from each treatment group) was extracted using the TRIzol reagent (Invitrogen, CA, United States). Specifically, DNase I was used during the RNA isolation process to remove contamination with genomic DNA. The quantity and purity of the total RNA was analyzed by a NanoDrop^®^ ND-1000 spectrophotometer (Thermo Scientific, MA, United States), and the integrity of the RNA was assessed with the Bioanalyzer 2100 and RNA Nano6000 LabChip Kit (Agilent, CA, United States). Only samples that had an OD260/280 > 1.8, OD260/230 > 2.0, and an RNA Integrity Number > 7.0 were used for further sequencing.

Approximately 3 μg of the total RNA from each sample was used to prepare an mRNA library according to the Illumina^®^ TruSeq^TM^ RNA sample preparation protocol. Then, the paired-end sequencing (2 × 125 bp) was performed on an Illumina HiSeq 2500. The 125 bp paired-end raw reads were first processed through SOAPnuke filter to obtain the clean data (Chen et al., 2017) by removing the reads that contain sequencing adapter contaminations or poly-N and the low-quality reads with *Q* values less than 20. At the same time, the Q20 and Q30 (note: Q20 and Q30 were widely used to adjust the quality of sequencing data. Q20 indicates the probability of an incorrect base call is 1 in 100, and Q30 indicates the probability of an incorrect base call is 1 in 1000.) of the clean data were calculated (Supplementary File 1: Table S1), and all of them indicated that our data were in good quality (with Q20 > 97% and Q30 > 92%). The index of the reference genome was built using Bowtie v2.2.3 (Langmead and Salzberg, 2012), and the sequences were aligned to the yak genome (*Bos mutus*, assembly BosGru_v2.0) using HISAT (Kim et al., 2015). Sequence segments were spliced and annotated, and transcript expressions were calculated by RSEM function (Li and Dewey, 2011). Fragments per kilobase of exon per million mapped reads (FPKM) was employed to quantify the gene expression. Based on negative binomial distribution, DEGs were screened out based on the expected read counts (fifth column of the output results) from the RSEM by using DESeq with an adjusted *P* < 0.05 and fold change > 2 or <0.5. In brief, the RSEM runs Bowtie to find all alignments of a read with at most two mismatches in its first 25 bases, to obtain the expected read count of each library. Then we combined differential comparison library to obtain the raw count matrix of gene expression. At last, the raw count matrix of gene expression was set as the raw count to DESeq2 to analyze and obtain the DEG (Li and Dewey, 2011). Kyoto Encyclopedia of Genes and Genomes (KEGG) pathway enrichment analysis for the DEGs was performed by using KOBAS 2.0 software (Xie et al., 2011). *P* < 0.05 was used to define KEGG pathways as significantly enriched.

### Quantitative Real-Time PCR (qRT-PCR) Analysis

Approximately 1 μg of total RNA was reverse transcribed using the PrimeScript^TM^ RT reagent Kit with gDNA eraser (TaKaRa, Dalian, China). qRT-PCR was performed using SYBR^®^ Green PCR Master Mix (TaKaRa, Dalian, China). A 20 μL PCR mixture was quickly prepared. Primers for *GAPDH* (internal control genes) and tested mRNAs [syndecan 4 (SDC4), selenoprotein W (SEPW1), Fos proto-oncogene (FOS), and FOS like 1 (FOSL1), which selected by RNA sequencing] were designed using Primer-BLAST^3^ and listed in Table 2. The PCR was conducted in an iCycler iQ5 multicolor real-time PCR detection system (Bio-Rad Laboratories) and programmed as follows: 95°C for 10 min; 40 cycles of 95°C for 10 s; 60°C for 30 s; 72°C for 30 s; and 72°C for 5 min. All the samples were examined in triplicate. All the data was analyzed using the 2^–ΔΔCt^ method (Livak and Schmittgen, 2001).

**TABLE 2:** Primer sequences for GAPDH (internal control genes) and tested mRNAs (DEGs selected by RNA sequencing).

Gene name/abbreviation	Primer sequences (5′to 3′)	Production size
*Glyceraldehyde-3-phosphate dehydrogenase GAPDH*	F: CGACTTCAACAGCGACACTCA R: GGTCCAGGGACCTTACTCCTT	160
*Syndecan 4 SDC4*	F: CTATGCAGAGAGGAGAGGCC R: AGAGGAAAAGGGACATGGGG	170
*Selenoprotein W SEPW1*	F: GGACACGGAGAGCAAGTTTC R: GAGATGAGGGATGGGGAAGG	288
*Fos proto-oncogene FOS*	F:TTTGACTGCTCGCGATCATG R:CAGATCGGTGCAGTAGTCCT	176
*FOS like 1 FOSL1*	F: CCCTGCTCATTTGATCCAGC R: GATTAGGGCTCCAGAGGACC	261

### Statistical Analysis

The statistical evaluation of the mRNA sequencing and 16S rRNA gene sequencing results were analyzed by using the bioinformatics methods described as above.

The statistical analyses on all the measures (except for the mRNA sequencing, 16S rRNA gene sequencing), including the mean of the individual feed intakes for the whole experimental period, were analyzed using the one-way ANOVA procedure by SPSS 21.0. If a significant treatment effect was indicated by ANOVA, the significance between the treatment differences was further identified by Duncan’s multiple comparisons test. All the data is expressed as the least square means with the standard error of the means. Differences were declared to be statistically significant at *P* < 0.05.

## Results

### Simultaneous Supplementation of Alfalfa and Starter Feeding Significantly Promoted the Growth of Yak Calves

**FIGURE 1:**
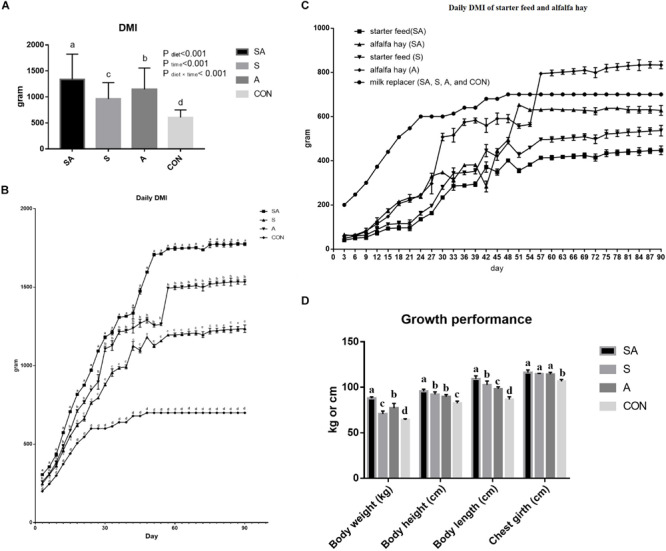
Effects of individual and simultaneous supplementation with alfalfa and starter feeding during pre-weaning period on average dry matter intakes (DMI) of overall experiment **(A)**, daily DMI of each measured day **(B)**, individual daily DMI of alfalfa hay and starter feed **(C)**, and body weight and body size traits **(D)** of yak calves. ^a–b^Different superscripts above the error bars for the same index indicated significant differences among different groups (*P* < 0.05); CON, supplemented with milk replacer only; S, supplemented with milk replacer and starter feed; A, supplemented with milk replacer and alfalfa hay; SA, supplemented with milk replacer, starter feed, and alfalfa hay.

The significant differences between the treatments in the daily DMI of the yak calves over the whole experimental period were identified in the present study, where the highest intake was found for calves in the SA group, followed with the S group, the A group, and the CON group, had the lowest feed intake (*P* < 0.05, Figure 1A). The DMIs of the calves were gradually increased with the gain of body weight of the calves, and the differences between the treatments in feed intake were persistent over the whole period (Figure 1B). Furthermore, among these three groups, which were supplied with starter feed or alfalfa hay, the intake of starter feed in the S group was significantly higher than the intake of starter feed in the SA group, and the intake of alfalfa hay in the A group was significantly higher than the intake of alfalfa hay in the SA group over the whole experimental period (Figure 1C).

Compared with the yak calves in the control group, significantly increased body weight, body height, body length, and chest girth were all identified in the 120-day-old yaks from the S, A, and SA groups (Figure 1D). Of these three groups, the yak calves from the SA group exhibited the most significantly improved growth performance based on the indexes of body weight, body height, body length, and chest girth when compared with those indexes in the yak calves from the A and S groups (Figure 1D).

### Simultaneous Supplementation of Alfalfa and Starter Feeding Significantly Enhanced the Ruminal Fermentation and Epithelium Development, and Increased Ruminal and Abomasal Enzymic Activities

The ruminal fermentation characteristics of yak calves of these four groups were significantly altered (Table 3). The supplementation with starter feeding (the S and SA groups) could significantly reduce the rumen pH compared with that of the CON and A groups. The supplementation with alfalfa and starter feeding could significantly increase the ruminal NH3-N concentration compared with the CON group; specifically, the individual supplementation of starter feeding (the S group) had the ruminal highest NH_3_-N concentration. The total VFA concentration was significantly higher in yak calves of the SA, S, and A groups when compared to the CON group, whereas yak calves in the SA group had the highest concentration of VFA, followed by the S group, and the A group was lower than the SA and S groups. Furthermore, those calves supplied with extra alfalfa hay, or starter feeding, or both alfalfa hay and starter feeding along with the milk replacer could significantly increase the butyrate, isobutyrate, acetate, and isovalerate contents compared with those contents in the CON group. Meanwhile, compared with the CON and A groups, the significantly increased ruminal propionate and valerate were identified in the calves from the SA and S groups. Of these, the supplementation of alfalfa could significantly increase the content of the acetate, and the supplementation of starter feeding could significantly increase the content of the propionate. Furthermore, compared with the CON group, the starter feeding supplementation could significantly increase the proportions of butyrate and isovalerate, and the alfalfa hay supplementation and the co-supplementation of starter and alfalfa hay could both significantly increase the proportions of acetate, butyrate, and isovalerate.

**TABLE 3:** Effects of individual and simultaneous supplementation with alfalfa and starter feeding on the rumen fermentation characteristics of yak calves.

Rumen fermentation indexes	Starter feeding + Alfalfa	Starter feeding	Alfalfa	CON	SEM	*P*-value
pH value	6.86^b^	6.90^b^	7.54^a^	7.68^a^	0.117	0.007
NH_3_-N (mg/dL)	6.91^b^	8.17^a^	6.40^b^	4.81^c^	0.315	<0.001
Acetate (mmol/L)	42.09^a^	34.47^c^	36.86^b^	16.31^d^	2.249	<0.001
Propionate (mmol/L)	11.53^b^	15.4^a^	8.49^c^	9.42^c^	0.626	<0.001
Isobutyrate (mmol/L)	1.56^a^	1.49^a^	1.16^b^	0.57^c^	0.099	<0.001
Butyrate (mmol/L)	8.60^a^	6.42^b^	5.21^c^	1.36^d^	0.623	<0.001
Isovalerate (mmol/L)	1.87^a^	2.11^a^	1.44^b^	0.61^c^	0.143	<0.001
Valerate (mmol/L)	1.18^a^	1.10^a^	0.81^b^	0.72^b^	0.060	0.005
Acetate/Total VFA	0.63^b^	0.57^c^	0.68^a^	0.56^c^	0.012	<0.001
Propionate/Total VFA	0.17^c^	0.25^b^	0.15^c^	0.32^a^	0.016	<0.001
Butyrate/Total VFA	0.13^a^	0.11^b^	0.10^b^	0.05^c^	0.007	<0.001
Isobutyrate/Total VFA	0.023	0.024	0.022	0.020	0.008	0.131
Valerate/Total VFA	0.018^b^	0.018^b^	0.015^b^	0.025^a^	0.001	0.001
Isovalerate/Total VFA	0.028^b^	0.035^a^	0.027^bc^	0.021^c^	0.001	0.002
Total VFA (mmol/L)	66.82^a^	60.99^b^	53.97^c^	28.98^d^	0.991	<0.001

*^*a*−*b*^ within a row with different superscripts means significantly difference which tested by One-way ANOVA test (*P* < 0.05); VFA, volatile fatty acid; NH3-N, ammoniacal nitrogen.*

**FIGURE 2:**
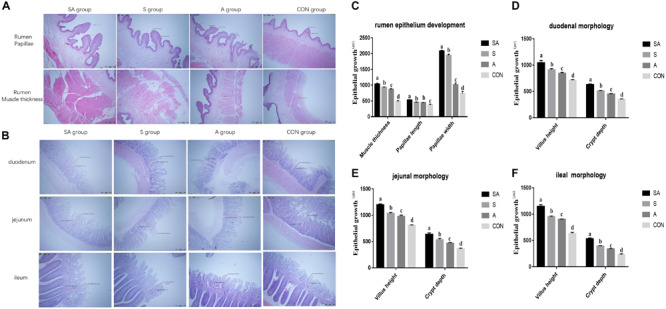
Effects of individual and simultaneous supplementation with alfalfa and starter feeding during the pre-weaning period on the ruminal **(A,C)** and intestinal morphology of yak calves **(B,D–F)**. ^a–d^Different superscripts above the error bars for the same index indicated significant differences among different groups (*P* < 0.05); CON, supplemented with milk replacer only; S, supplemented with milk replacer and starter feed; A, supplemented with milk replacer and alfalfa hay; SA, supplemented with milk replacer, starter feed, and alfalfa hay.

Furthermore, rumen epithelium development was also measured (Figures 2A,C). The papillae length and width of rumen epithelium and the thickness of the rumen base were all significantly increased in yak calves, which were individually or simultaneously fed with alfalfa and starter feeding, and the rumen epithelium development of the yak calves in the SA group was the most significantly enhanced (*P* < 0.001). Meanwhile, compared with the A group, the significantly increased papillae length of rumen epithelium and the thickness of the rumen base were identified in the S group, whereas the co-supplementation with alfalfa and starter feeding was beneficial to the rumen epithelium development compared with the S and A groups (*P* < 0.001).

**FIGURE 3:**
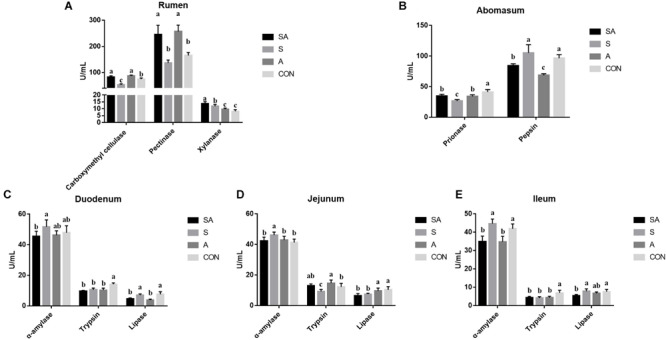
Effects of individual and simultaneous supplementation with alfalfa and starter feeding during the pre-weaning period on the ruminal **(A)**, abomasum **(B)**, and intestinal enzymic activities **(C–E)** of yak calves. ^a–c^Different superscripts above the error bars for the same index indicated significant differences among different groups (*P* < 0.05); CON, supplemented with milk replacer only; S, supplemented with milk replacer and starter feed; A, supplemented with milk replacer and alfalfa hay; SA, supplemented with milk replacer, starter feed, and alfalfa hay.

Meanwhile, the ruminal enzymic activities were also tested. Compared with the CON group, the carboxymethyl cellulase activity was significantly increased in the SA and A groups, whereas it was decreased in the S group. Meanwhile, the supplementation of alfalfa (the SA and A groups) could increase the pectinase activity, and the supplementation with the starter feeding (the SA and S groups) could increase the xylanase activity (Figure 3A). In the abomasum, the pepsase activity was significantly decreased in the S, A, and SA groups compared with the CON group, and the chymosin activity in the SA and A groups was significantly decreased compared with the CON and S groups (Figure 3B).

### Simultaneous Supplementation of Alfalfa and Starter Feeding Significantly Increased Intestinal Enzymic Activities and Promoted Intestinal Epithelium Development

The intestinal morphology of the yak calves was measured (Figures 2B,D–F). In the S, A, and SA groups, the villus height and crypt depth of the duodenum, jejunum, and ileum were significantly increased compared with those measures in the CON groups (*P* < 0.001). Among the SA, S, and A groups, the simultaneous supplementation of alfalfa and starter feeding could significantly promote the development of intestinal villus height and crypt depth (Figures 2B,2D–F).

The intestinal digestion functions of yak calves from the four groups were further examined (Figure 3). In the intestine (Figures 3C–E), when the starter feeding group (S group) was compared with the three other groups, the α-amylase activity of the duodenum, jejunum, and ileum was significantly increased in the yak calves. Compared with the CON group, the S, A, and SA groups, except for showing increased trypsin activity in the jejunum, showed significantly decreased trypsin activity with the individual or simultaneous supplementations. Meanwhile, the simultaneous supplementation of the alfalfa and starter feeding could significantly decrease the lipase activity of the duodenum, jejunum, and ileum compared with the CON group.

### Simultaneous Supplementation of Alfalfa and Starter Feeding Significantly Enhanced Health Condition of Yak Calves

The immune- and antioxidant-capacity-related indexes of blood and plasma, including ALT, AST, AST/ALT, ALP, TP, ALB, GLO, ALB/GLO, LDH, IgG, NO, lysozyme, TNFα, IFN-γ, IL-1β, IL-2, IL-4, IL-10, and alexine C3, were established to determine the health conditions of yak calves from the four groups (Table 4). As a result, the individual or simultaneous supplementation of starter feeding and alfalfa was able to significantly decrease the AST/ALT and increase the content of ALB. Furthermore, the content of NO in the S group was significantly increased compared with that in the other three groups, and the content of TNF-α in the A group was significantly decreased compared with that in the CON and S groups. The IFN-γ contents in the SA and S groups were significantly increased compared with that in the CON and A groups. Meanwhile, the co-supplementation with alfalfa and starter feeding could significantly decrease the lysozyme content compared with that of the CON group.

**TABLE 4:** Effects of individual and simultaneous supplementation with alfalfa and starter feeding on the plasma indexes related to immune conditions of yak calves.

Plasma indexes	Starter feeding + Alfalfa	Starter feeding	Alfalfa	CON	SEM	*P*-value
ALT (U/L)	29.2	26.4	30.8	22.4	1.26	0.081
AST (U/L)	85.0	88.2	87.4	102.6	3.86	0.383
AST/ALT	2.96^b^	3.36^b^	2.86^b^	4.57^a^	0.177	<0.001
ALP (U/L)	144.1^a^	121.4^bc^	126.4^b^	111.5^c^	3.34	0.001
TP (g/L)	62.86	62.24	66.30	60.04	1.12	0.274
ALB (g/L)	36.24^a^	35.70^a^	36.06^a^	30.98^b^	0.65	0.002
GLO (g/L)	26.62	26.54	30.24	27.26	0.74	0.252
A/G	1.38	1.36	1.22	1.16	0.038	0.113
LDH (IU/L)	1249.40	1308.80	1201.60	1143.60	41.224	0.572
IgG (g/L)	12.99	12.91	11.89	12.50	0.284	0.536
NO (μmol/mL)	49.21^b^	58.28^a^	48.03^b^	49.88^b^	1.177	0.001
Lysozyme (μg/mL)	3.44	3.82	3.72	3.92	0.078	0.130
TNF-α (pg/mL)	72.30^ab^	74.37^a^	69.91^b^	73.01^a^	0.57	0.027
IFN-γ (pg/mL)	337.58^a^	323.55^b^	282.57^c^	278.25^c^	5.99	<0.001
IL-1β (pg/mL)	128.33	127.62	136.97	134.93	1.70	0.120
IL-2 (pg/mL)	152.85	152.01	159.80	161.76	1.79	0.122
IL-4 (pg/mL)	20.84	21.14	17.80	18.48	0.61	0.122
IL-10 (pg/mL)	23.32	23.56	20.89	21.44	0.49	0.120
Alexine C3 (g/L)	0.672	0.666	0.726	0.760	0.019	0.250

*^*a*−*c*^ within a row with different superscripts means significantly difference which tested by One-way ANOVA test (*P* < 0.05); ALT, alanine aminotransferase; AST, aspartate aminotransferase; ALP, alkaline phosphatase; TP, total protein; ALB, albumin; GLO, globulin; LDH, lactic dehydrogenase; IgG, immune globulin G; NO, nitric oxide; TNF-α, tumor necrosis factor α; IFN-γ, interferon γ; IL, interleukin.*

**FIGURE 4:**
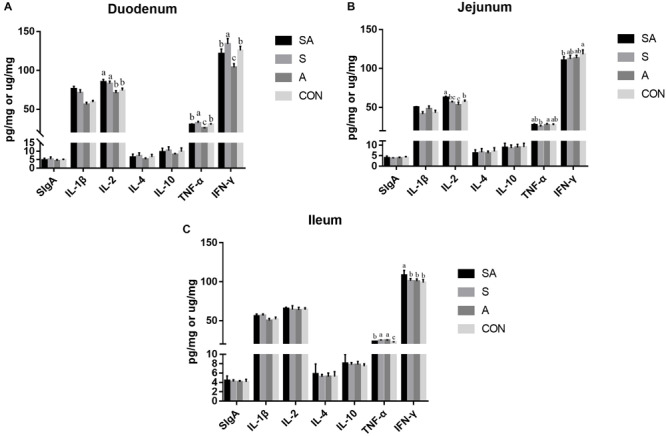
Effects of individual and simultaneous supplementation with alfalfa and starter feeding during the pre-weaning period on the sIgA and immune cell cytokine content of mucous membrane samples of the duodenum **(A)**, jejunum **(B)**, and ileum **(C)**. ^a–c^Different superscripts above the error bars for the same index indicated significant differences among different groups (*P* < 0.05); CON, supplemented with milk replacer only; S, supplemented with milk replacer and starter feed; A, supplemented with milk replacer and alfalfa hay; SA, supplemented with milk replacer, starter feed, and alfalfa hay.

The intestinal immune functions were further evaluated. In the present study, the sIgA, IL-1β, IL-2, IL-4, IL-10, TNF-α, and IFN-γ contents of the mucous membrane samples of the duodenum, jejunum, and ileum were first measured by ELISA (Figure 4). In the duodenum, the IL-1β and IL-2 contents were significantly increased in the SA and S groups compared with those of the A and CON groups, and the contents of the TNF-α and IFN-γ were significantly increased in the S group while being decreased in the A group. In the jejunum, the IL-1β and IL-2 contents were significantly increased in the SA groups compared with those in the S and CON groups; meanwhile, the IL-1β content was significantly increased in the A group compared with that of the CON group, whereas the content of IL-2 was significantly decreased in the A group compared with that of the CON group. Furthermore, the TNF-α content in the A group was significantly increased compared with that in the S group, and the IFN-γ content in the SA group was significantly decreased compared with that in the CON group. In the ileum, the IL-1β content was significantly increased in the SA and S groups compared with that in the A and CON groups, and the IFN-γ content was significantly increased in the SA group compared with that in the S, A, and CON groups. Meanwhile, the TNF-α contents in the SA, S, and A groups were significantly increased compared with that in the CON group, whereas the TNF-α content in the SA group was significantly decreased compared with that in the S and A groups.

**FIGURE 5:**
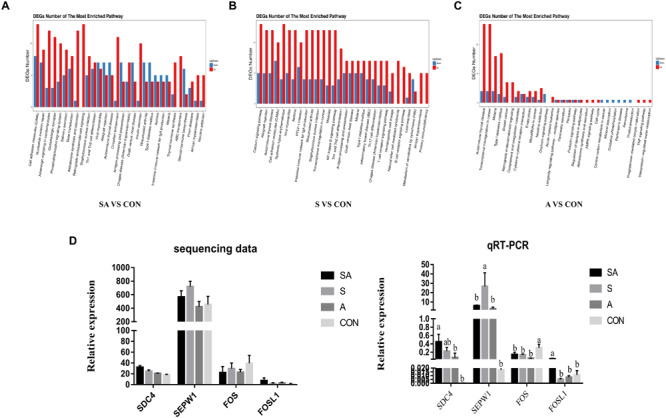
Differentially expressed genes (DEGs) of rumen epithelium between the yak calves induced by individual and simultaneous supplementation with alfalfa and starter feeding. **(A–C)** The top 30 mostly significantly enriched pathways based on the identified DEGs of different compared groups includes SA vs. CON, S vs. CON, and A vs. CON; **(D)** validation of the accuracy of RNA-seq data by qRT-PCR. CON, supplemented with milk replacer only; S, supplemented with milk replacer and starter feed; A, supplemented with milk replacer and alfalfa hay; SA, supplemented with milk replacer, starter feed, and alfalfa hay. ^a,b^within a row with different superscripts means significantly difference.

RNA sequencing was performed to study the effects of individual or simultaneous supplementation of starter feeding and alfalfa on the mRNA profiles of rumen epithelial tissue (Figure 5). In brief, based on 45.8 million of average obtained reads and the 42.7 million of average clean reads (Supplementary File 1: Table S1), 812, 1023, 1877, 532, 930, and 1194 DEGs were identified in compared groups as follows: SA vs. S, SA vs. A, SA vs. CON, S vs. A, S vs. CON, and A vs. CON (Figure 5A), and the count of each compared DEGs of different group is shown in Supplementary File 2: Table S1. The qRT-PCR verification analyses proved that the transcriptomic analyses were reproducible and reliable (Figure 5D). Based on these identified DEGs, the KEGG enrichment analyses further identified 57, 53, and 64 significantly enriched KEGG pathways (Supplementary File 1: Table S2) based on the DESs from the compared groups of SA vs. CON, S vs. CON, and A vs. CON (the 30 most significantly enriched pathways were shown as Supplementary File 1: Figure S1), respectively, and most of these significantly enriched pathways were related to the immune regulation process. Accordingly, our results indicated that the individual or simultaneous supplementation with starter feeding and alfalfa could significantly enhance the ruminal immune function by regulating the NF-kappa B signaling pathway, the intestinal immune network for IgA production, Th1 and Th2 cell differentiation, inflammatory bowel disease (IBD), the T cell receptor signaling pathway, Th17 cell differentiation, the B cell receptor signaling pathway, and the natural killer cell–mediated cytotoxicity pathway (Figure 5B). Meanwhile, we found that the supplementation with starter feeding, which acted as the grain feed in the ruminal diets, contributed more to regulate the immune function by influencing the gene expression involved in the intestinal immune network for IgA production, the NF-kappa B signaling pathway, inflammatory bowel disease (IBD), Th1 and Th2 cell differentiation, Th17 cell differentiation, the T cell receptor signaling pathway, the B cell receptor signaling pathway, and the natural killer cell–mediated cytotoxicity pathway (Figure 5C). Comparably, supplementation with alfalfa, which acted as roughage in the ruminal feed, could regulate the immune function by influencing the genes involved in the TNF signaling pathway and the Cytokine-cytokine receptor interaction pathway (Figure 5D).

### Significantly Altered Gastrointestinal Microbiota Induced by Simultaneous Supplementation of Alfalfa and Starter Feeding

**FIGURE 6:**
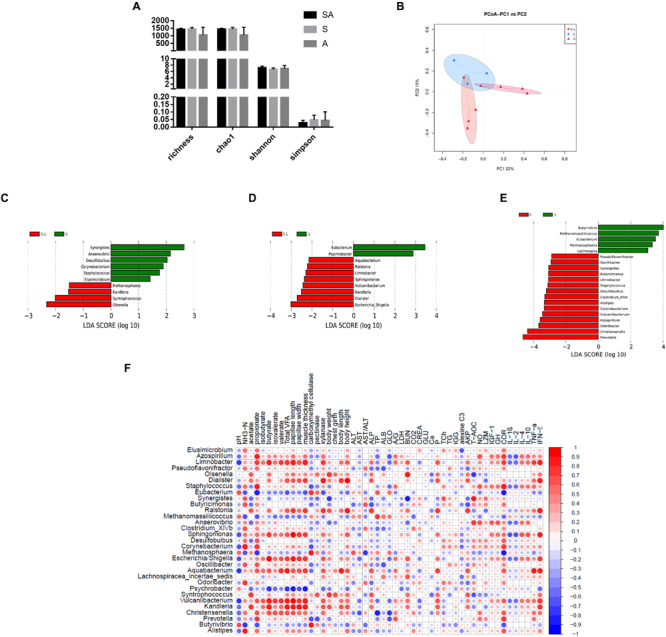
The rumen microbial community differences induced by the individual and simultaneous supplementation with alfalfa and starter feeding. **(A)** α-diversity analyses based on the rumen microbiota; **(B)** β-diversity analyses based on the rumen microbiota; **(C)** significantly increased genera in SA group (red bar) and S group (green bar) in compared group of SA vs. S; **(D)** significantly increased genera in SA group (red bar) and A group (green bar) in compared group of SA vs. A; **(E)** significantly increased genera in S group (red bar) and A group (green bar) in compared group of S vs. A; **(F)** significantly Spearman correlation between the identified differential ruminal genera and the significantly altered growth performance, healthy condition, ruminal enzymic activities, and ruminal development of yak calves. Blue block indicated negative correlation and red block indicated positive correlation. CON, supplemented with milk replacer only; S, supplemented with milk replacer and starter feed; A, supplemented with milk replacer and alfalfa hay; SA, supplemented with milk replacer, starter feed, and alfalfa hay.

**FIGURE 7:**
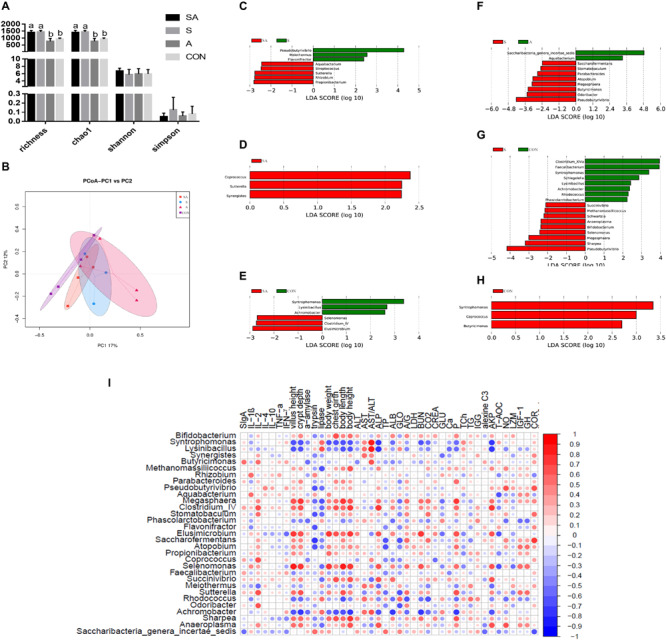
The jejunal microbial community differences induced by the individual and simultaneous supplementation with alfalfa and starter feeding. **(A)** α-diversity analyses based on the rumen microbiota; **(B)** β-diversity analyses based on the rumen microbiota; **(C)** significantly increased genera in SA group (red bar) and S group (green bar) in compared group of SA vs. S; **(D)** significantly increased genera in SA group (red bar) in compared group of SA vs. A; **(E)** significantly increased genera in SA group (red bar) and CON group (green bar) in compared group of SA vs. CON; **(F)** significantly increased genera in S group (red bar) and A group (green bar) in compared group of S vs. A; **(G)** significantly increased genera in S group (red bar) and CON group (green bar) in compared group of S vs. CON; **(H)** significantly increased genera in CON group (red bar) in compared group of A vs. CON; **(I)** Significantly Spearman correlation between the identified jejunal differential genera and the significantly altered growth performance, healthy condition, and jejunal enzymic activities, and development of yak calves. Blue block indicated negative correlation and red block indicated positive correlation. CON, supplemented with milk replacer only; S, supplemented with milk replacer and starter feed; A, supplemented with milk replacer and alfalfa hay; SA, supplemented with milk replacer, starter feed, and alfalfa hay. ^a,b^within a row with different superscripts means significantly difference.

**FIGURE 8:**
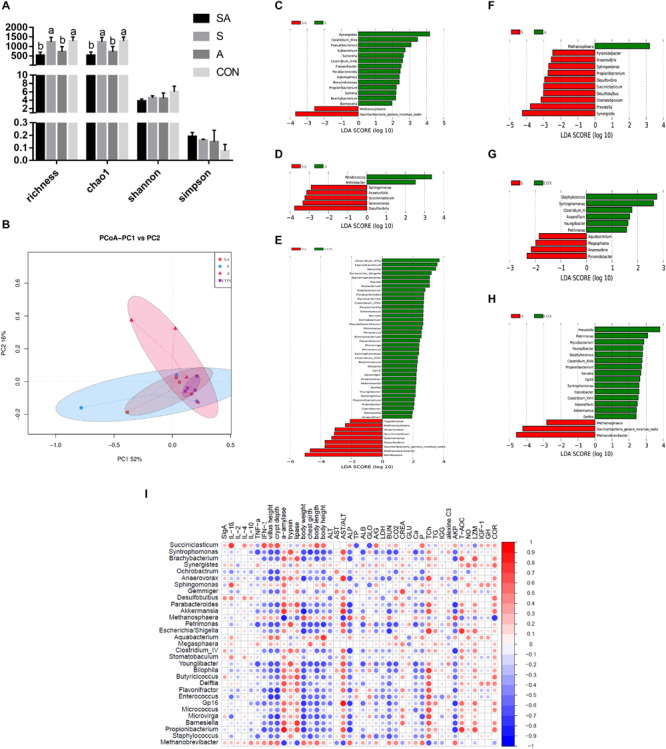
The ileal microbial community differences induced by the individual and simultaneous supplementation with alfalfa and starter feeding. **(A)** α-diversity analyses based on the rumen microbiota; **(B)** β-diversity analyses based on the rumen microbiota; **(C)** significantly increased genera in SA group (red bar) and S group (green bar) in compared group of SA vs. S; **(D)** significantly increased genera in SA group (red bar) and A group (green bar) in compared group of SA vs. A; **(E)** significantly increased genera in SA group (red bar) and CON group (green bar) in compared group of SA vs. CON; **(F)** significantly increased genera in S group (red bar) and A group (green bar) in compared group of S vs. A; **(G)** significantly increased genera in S group (red bar) and CON group (green bar) in compared group of S vs. CON; **(H)** significantly increased genera in A group (red bar) and significantly increased genera in CON group (green bar) in compared group of A vs. CON; **(I)** Significantly Spearman correlation between the identified ileal differential genera and the significantly altered growth performance, healthy condition, and ileal enzymic activities and development of yak calves. Blue block indicated negative correlation and red block indicated positive correlation. CON, supplemented with milk replacer only; S, supplemented with milk replacer and starter feed; A, supplemented with milk replacer and alfalfa hay; SA, supplemented with milk replacer, starter feed, and alfalfa hay. ^a,b^within a row with different superscripts means significantly difference.

The gastrointestinal microbiota, including the microbiota in the rumen, jejunum, and ileum, were further analyzed. According to the alpha diversity analyses, chao1 indexes indicated that the SA and S groups were both beneficial to the diversity of the jejunal microbiota when compared with the A and CON groups (Figures 6A, 7A). Meanwhile, the S and CON groups could significantly increase the Chao index of ileal microbiota when compared with the A and SA groups (Figure 8A). Moreover, the beta diversity analyses revealed that the compositions of the gastrointestinal prokaryotic community of the yak calves in different feeding groups were significantly different (*P* < 0.05) in rumen, jejunum, and ileum (Figures 6B, 7B, 8B).

Based on the identified microbiota of the rumen, jejunum, and ileum, the differential genera based on the relative abundance of genera were further identified by using LEfSe analyses (Figures 6–8). In the present study, we failed to identify the rumen microbiota of calves from the CON group. Herein, we only identified the differentially abundant microbiota among the another three groups, and 10, 10, and 20 differentially abundant genera were respectively identified based on the group comparisons of SA vs. S, SA vs. A, and S vs. A (Figures 6C–E). On this basis, the Spearman correlation between the identified differential genera and the significantly changed performance indexes were further tested (Figure 6F). Of these, in the compared group of SA vs. S, the significantly increased relative abundances of *Kandleria*, *Syntrophococcus*, and *Olsenella* in the SA group were significantly positively correlated with the increased growth performance, ruminal fermentation parameters, ruminal enzymic activities, and ruminal epithelium development. Similarly, in the compared group of SA vs. A, the significantly increased relative abundances of *Escherichia*/*Shigella*, *Dialister*, *Kandleria*, *Vulcaniibacterium*, *Sphingomonas*, *Limnobacter*, *Ralstonia*, and *Aquabacterium* in the SA group were significantly positively correlated with the increased growth performance, ruminal fermentation parameters, ruminal enzymic activities, and ruminal epithelium development as well, but the increased *Limnobacter*, *Escherichia*/*Shigella*, and *Aquabacterium* were positively correlated with the IFN-γ content but negatively correlated with the IL-1β and TNF-α contents (Figure 6F and Supplementary File 3: Table S1).

In the jejunum, 8, 3, 6, 10, 17, and 3 differential microbiota were identified in six group comparisons, including SA vs. S, SA vs. A, SA vs. CON, S vs. A, S vs. CON, and A vs. CON (Figures 7C–H). Of these, the significantly increased relative abundances of *Elusimicrobium*, *Clostridium IV*, and *Selenomonas* in the SA group when compared with the S, A, and CON groups were significantly positively correlated with the increased growth performance, jejunal enzymic activities, and jejunal epithelium development, while the significantly increased relative abundances of *Coprococcus*, *Pseudobutyrivibrio*, *Flavonifractor, Synergistes*, and *Sutterella* were positively correlated with the content of anti-inflammatory cytokines or negatively correlated with the content of pro-inflammatory cytokines (Figure 7I and Supplementary File 3: Table S1).

Similarly, in the ileum, 16, 7, 47, 11, 10, and 18 differential microbiota were further identified in the six group comparisons (Figures 8C–H). Of these, the significantly increased relative abundances of *Akkermansia*, *Anaerovibrio*, *Anaerovorax*, *Barnesiella*, *Akkermansia*, *Bilophila*, *Blautia*, *Brachybacterium*, *Butyricicoccus*, *Clostridium_XlVb*, *Coprobacter*, *Delftia*, *Desulfovibrio*, *Enterococcus*, *Eubacterium*, *Flavonifractor*, *Gp16*, *Haemophilus*, *Methanosphaera*, *Micrococcus*, *Microvirga*, *Mycobacterium*, *Parabacteroides*, *Paracoccus*, *Parasutterella*, *Petrimonas*, *Propionibacterium*, *Pseudoflavonifractor*, *Rubrobacter*, *Saccharibacteria genera incertae sedis*, *Selenomonas*, *Serratia*, *Sphingomonas*, *Staphylococcus*, *Stenotrophomonas*, *Succiniclasticum*, *Syntrophomonas*, and *Youngiibacter* in the SA group, when compared with the S, A, and CON groups were significantly positively correlated with the increased growth performance, jejunal enzymic activities, or jejunal epithelium development (Figure 8I and Supplementary File 3: Table S1).

## Discussion

### Simultaneous Supplementation of Alfalfa and Starter Feeding Was Mostly Beneficial to Promote the Growth and Improve Immune Functions of Yak Calves

Early dietary experiences, especially supplementation with carbohydrate nutrition, during the pre-weaning period have crucial long-term impacts on enhanced ruminal fermentation and gastrointestinal immune function in ruminants (Liu et al., 2017; Yang et al., 2018). The pre-weaning period for yak calves mainly occurs during maternal grazing and nursing, which lasted 120–180 days on the Qinghai-Tibetan Plateau. However, the calves by maternal grazing and nursing could not obtain enough nutrients growth. Barn feeding and early weaning with alfalfa hay and starter feeding can be beneficial to the growth and carcass characteristics of ruminants (Zi et al., 2004; Dong et al., 2006; Chen et al., 2015) and can serve as an alternative to the maternal grazing for yak calves. Hence, with aims to screen out the suitable early weaning paradigms, the present study compared different feeding methods of yak calves during the pre-weaning period, which were separately fed with the milk replacer, milk replacer with alfalfa hay, milk replacer with starter feeding, and milk replacer with alfalfa and starter feeding. Based on the observations that we observed significantly enhanced gastrointestinal development, digestion-absorption, and immune function of the calves from the SA group, the simultaneous supplementation with alfalfa and starter feeding could significantly improve the growth performance and immune function of yak calves and could serve as the optimal early weaning paradigms for yak calves. In the previous studies, the roles of individual supplementation with alfalfa and starter feeding in regulating the rumen functions of calves or lambs have been widely suggested (Liu et al., 2017; Yang et al., 2018). The supplementation of starter feeding during pre- or post-weaning has been widely used to feed young pre-weaned ruminants, mainly due to its ability to promote rumen development by enhancing rumen fermentation and VFA production, primarily propionate production (Jiao et al., 2015; Wang et al., 2016), which could provide more energy to the development of the rumen and the intestine. Moreover, the supplementation of starter feeding also proved to modulate colonic mucosal and ruminal immune homeostasis in the weaning of lambs or calves, which is due in part to the enrichment of some beneficial bacteria and the depression of some pathogenic bacteria during starter feeding in lambs (Liu et al., 2017). Moreover, a starter diet containing alfalfa could enhance rumen fermentation and VFA production (Baldwin et al., 2012); promote ruminal muscular development and size expansion; increase the rumen papillae length and the rumen weight; decrease the incidence of feed plaques and the inflammation of the rumen; and, consequently, lead to an increased feed intake, an average daily gain (ADG), and carcass weight during the pre- and post-weaning periods (Yang et al., 2015, 2018). Overall, these previous results have all indicated that individual or simultaneous supplementation with alfalfa and starter feeding are beneficial to the gastrointestinal nutrient utilization and immune homeostasis, further promote the growth and development, and enhance the immune function of calves or lambs, which are in accordance with the findings in the present study. And mostly, the simultaneous supplementation with alfalfa and starter feeding could obtain the most optimal growth performance and immune condition.

### Alfalfa and Starter Feeding Significantly Promoted the Growth and Improved Gastrointestinal Immune Functions of Yak Calves in Differential and Complementary Ways

Most previous studies illuminated the beneficial effect of starter feeding or alfalfa hay on the immune function and the growth performance by adding extra starter feeding or alfalfa hay (Yang et al., 2015; Liu et al., 2017; Wang et al., 2017), but their regulatory mechanisms were rarely compared. With aims to illuminate how the simultaneous supplementation with alfalfa and starter feeding could obtain the optimal growth performance and immune condition, the present study compared the differences between individual or simultaneous supplementation with alfalfa and starter feeding and also determined that the improved intestinal development, digestion-absorption, and immune functions in the yak calves fed with extra alfalfa hay, or starter feeding, or both alfalfa hay and starter feeding along with the milk replacer. As results, we found that the roles of these supplemental alfalfa hay and starter feeding in regulating the growth performance and the immune function were not the same. With respect to growth performance, the significantly increased DMI of alfalfa or starter feed and the consequently increased rumen VFA production of the propionate, acetate, and butyrate were identified after starter feeding and alfalfa hay supplementation, and this could be the main reason for the enhanced growth performance (Khan et al., 2008). The alfalfa and the starter feeding were both able to significantly enhance the rumen fermentation, whereas the alfalfa was beneficial to the acetic fermentation, and the starter feeding was beneficial to the propionic fermentation, which could be separated into fat and glucose to provide energy and promote the growth of the yak calves (Beauchemin et al., 2003; Khan et al., 2008; Laarman et al., 2012). Meanwhile, the butyrate could serve as a direct energy source for the gastrointestinal epithelial cells and promote rumen and intestinal development (Guilloteau et al., 2010; Baldwin et al., 2012). The supplementation with the starter feeding could also increase the butyrate fermentation, which served as the main cause of improved rumen and intestinal development of the yak calves of the SA and S groups in the present study. Moreover, different from the supplemental alfalfa, the starter feeding supplementation indicated that more starch could pass into the small intestines, which further induced an increase in the intestinal amylase activity in the S and SA groups and were beneficial to the intestinal digestive ability of these groups (Khan et al., 2007; Kosiorowska et al., 2011). In combination, the alfalfa and the starter feeding, when co-supplemented, could improve the rumen fermentation and intestinal digestion-absorption function by obtaining the advantages gained from the individual supplementation with both the alfalfa and the starter feeding.

Regarding the enhanced immune function, although the supplementation with alfalfa and starter feeding both improved the gastrointestinal immune functions and further improved the health of the yak calves, their roles in improving the gastrointestinal immune function were also not the same. Based on the KEGG analyses of the rumen epithelial DEGs, the extra alfalfa supplement could reduce the gene expressions in the TNF signaling pathway and the cytokine–cytokine receptor interaction pathway, which has been proven to be related to the occurrence of rumen inflammation (Leonard and Lin, 2000; Blaser et al., 2016). However, the starter feeding supplementation was mainly increase the gene expression in the Th1 and Th2 cell differentiation, the T cell receptor signaling pathway, Th17 cell differentiation, the B cell receptor signaling pathway, and the natural killer cell mediated cytotoxicity pathway, and these pathways were proved to be beneficial to the differentiation of immune cells and the improved immune function (Romagnani, 1992; Weaver et al., 2006). Furthermore, according to the expression of intestinal inflammatory cytokines, the supplementation with starter feeding could increase the expression of some proinflammatory cytokines, including the IL-2, TNF-α, and IFN-γ (Maggioli et al., 2016), which indicated that the grain feed supplementation could increase the occurrence of intestinal inflammation, stimulate the differentiation of immune cells, and enhance the immune function. Comparably, the alfalfa hay could reduce the expression of inflammatory cytokines and modulate the intestinal immune homeostasis in pre-weaning yaks. In combination, the starter feeding and alfalfa, when co-supplemented, could enhance the immune function and reduce the gastrointestinal inflammatory response, which was mostly beneficial to the intestinal immune homeostasis and the health of the yak calves. In summary, the complementary roles of alfalfa and starter feeding in regulating rumen fermentation and gastrointestinal immune functions ensure that simultaneous supplementation with alfalfa and starter feeding during the pre-weaning period could obtain the best growth performance and healthy condition of the yak calves.

### Altered Gastrointestinal Microbiota in Response to Alfalfa and Starter Feeding Intervention Contribute to Improved Gastrointestinal Nutrient Utilization and Immune Homeostasis in Yak Calves

Recent studies have suggested that the promotion of the establishment of different microbial populations would be possible in the rumen and the intestine by manipulating the feed supplements used during the weaning period, which could further alter the growth and the immune function of the young ruminants (Yáñez-Ruiz et al., 2015). Unfortunately, we failed to identify the rumen microbiota of yak calves from the CON groups, which probably induced by the limited rumen fluid samples obtained and the lower abundance of rumen microbiota in the CON group. These results may be induced by the esophageal groove reflex of yak calves in CON group, so that most of the milk replacer fluid does not pass through the rumen and directly pass into the abomasum (Orskov and Benzie, 1969). Moreover, although the appearance of the microbial populations precedes rumen development, it has been suggested that the development of the rumen and its microbiota begin with the intake of solid feed (Rey et al., 2014). Hence, based on the successfully sequenced microbiota, the significantly altered microbiota among different compared groups were identified. Furthermore, the Spearman correlation between the identified differential genera and the significantly changed performance indexes were further tested.

Of those genera that positively correlated to the promoted growth performance, a significantly increased microbe response was observed in response to the utilization of starch and the generation of propionate or butyrate or to the utilization of the lactic acid, which was induced by the starter feeding supplementation were identified in the SA group. For instance, the Pseudobutyrivibrio, Clostridium IV, and Desulfovibrio could generate butyrate by utilizing the starch (Kopecný et al., 2003; Balamurugan et al., 2008; Wang et al., 2013), and the Selenomonas, Brachybacterium, and Haemophilus could mainly take part in the propionate production process by utilizing the non-fibrous carbohydrates (Michel and Macy, 1990; Müller et al., 2010). Moreover, the abundance of Coprococcus, Sphingomonas, and Dialister has been widely suggested to be positively correlated with the increased starch supplementation, which suggested that these three genera could take part in the utilization of starch (Khafipour et al., 2009; Granja-Salcedo et al., 2017; Zhang et al., 2017). Meanwhile, the cellulose-decomposing and hemicellulose-decomposing related bacteria were also significantly increased in the yak calves from the SA group compared with those observed in the non-alfalfa supplemented groups (the CON or S group). For instance, the Eubacterium, Propionibacterium, Saccharibacteria genera incertae sedis, and Anaerovibrio have been proved to utilize fibrous carbohydrates and then mainly generate acetate (Bi et al., 2018; Xie et al., 2018; Yang et al., 2018), which could metabolize into fatty acids and further provide energy to the yak calves as well. In summary, the significantly increased microbes response to using different carbohydrates includes starch and fibrous carbohydrates were simultaneously identified in the SA group, which could further produce different VFAs and further benefit the development and digestion-absorption function of the rumen and intestine.

Moreover, the different microbes in the different groups could also influence the intestinal immune function in the present study. Of those identified microbes that were differentially present among the groups, the significant decrease in *Sutterella* in the alfalfa supplement groups (the SA and A groups) was proven to be negatively correlated with gastrointestinal inflammation (Mukhopadhya et al., 2011), which was consistent with the significantly decreased inflammation observed in the rumen and intestine in the present study. Meanwhile, the significant increase in *Oscillibacter*, *Parabacteroides*, *Bifidobacterium*, and *Blautia* in the starter feeding groups were proven to stimulate the immune response and enhance immune functions (O’Mahony et al., 2005; Kverka et al., 2011; Loh and Blaut, 2012), which were consistent with the significantly promoted differentiation of immune cells and significantly improved immune function of the starter feeding groups in the present study. Overall, our study has proven that the altered gastrointestinal microbiota responds to alfalfa and starter feeding intervention contributes to the improved gastrointestinal nutrient utilization and immune homeostasis in yak calves.

## Conclusion

Our study shows that alfalfa and starter feeding supplementations during the pre-weaning period are beneficial to the gastrointestinal development and its digestion, absorption, and immune function, as well as the subsequently enhanced growth and immune function of yak calves, which might benefit from the increased abundances of different genera that could use different carbon and nitrogen sources from both fibrous carbohydrate and non-fibrous carbohydrate. Overall, our findings suggest that after colostrum intake, a milk replacer and *ad libitum* starter feeding supplemented with alfalfa are recommended for the pre-weaning system to improve yak calf health and growth performance.

## Data Availability Statement

The raw data supporting the conclusions of this article will be made available by the authors, without undue reservation, to any qualified researcher. The sequence data were deposited and are available in the Sequence Read Archive (SRA) of NCBI with the accession project numbers PRJNA543073.

## Ethics Statement

This study was carried out in accordance with the recommendations of the Administration of Affairs Concerning Experimental Animals (Ministry of Science and Technology, China, revised 2004). The protocol was approved by the Institutional Animal Care and Use Committee of the Northwest A&F University (protocol number NWAFAC1118).

## Author Contributions

ZC, SW, SL, and JY conceived and designed the experiments. ZC, SW, SL, JL, Q-EY, SC, LW, XW, and XZ mainly performed the experiments. ZC and SW analyzed the data. JY and SL contributed the reagents, materials, and analysis tools, had primary responsibility for final content. SW and ZC wrote the manuscript. All authors read and approved the final manuscript.

## Conflict of Interest

The authors declare that the research was conducted in the absence of any commercial or financial relationships that could be construed as a potential conflict of interest.

## Footnotes

1 http://drive5.com

2 http://scotty.genetics.utah.edu/

3 http://www.ncbi.nlm.nih.gov/tools/primer-blast/

## Supplementary Material

FILE 1: FIGURE S1 The numbers of identified up-regulated and down-regulated DEGs of differential compared groups (SA vs. S, SA vs. A, SA vs. CON, S vs. A, S vs. CON, and A vs. CON).

FILE 1: TABLE S1 Statistical data and quality controlled data of the RNA-Sequencing reads for six samples.

FILE 1: TABLE S2 The significantly enriched pathways based on the identified DEGs of different compared groups (SA vs. CON, S vs. CON, and A vs. CON).

FILE 2: TABLE S3 The details of the differentially expressed genes and the count of each compared differentially expressed genes of different group (SA vs. S, SA vs. A, SA vs. CON, S vs. A, S vs. CON, and A vs. CON).

FILE 3: TABLE S4 The identified differential ruminal, jejunal, ileal genera which significantly correlated with the altered growth performance, healthy condition, ruminal enzymic activities, and ruminal development of yak calves based on the spearman correlation analyses.

## Abbreviations

ALB: albumin
ALP: alkaline
ALT: glutamic-pyruvic transaminase
AST: glutamic oxalacetic transaminase
DEGs: differentially expressed genes
FPKM: Fragments per kilobase of exon per million mapped reads
GLO: globulin
IFN- γ: interferon- γ
IgA/IgG/IgM: immunoglobulin
A/G/M IL-1 β: interleukin-1 β
IL-2: interleukin-2
IL-4: interleukin-4
IL-10: interleukin-10
KEGG: Kyoto Encyclopedia of Genes and Genomes
LDA: linear discriminant analysis
LDH: lactic dehydrogenase phosphatase
LEfSe: linear discriminant analysis effect size
NO: nitric oxide
OTUs: operational taxonomic units
PCoA: principal coordinated analysis
qRT-PCR: quantitative real-time PCR
sIgA: secreted immunoglobulin A
TNF- α: tumor necrosis factor- α
TP: total protein
VFA: volatile fatty acid.

## References

[B1] AOAC International (2000). *Official Methods of Analysis. 17th ed*. Arlington, VA: AOAC International.

[B2] Balamurugan R. Rajendiran E. George S. Samuel G. V. Ramakrishna B. S. (2008). Real-time polymerase chain reaction quantification of specific butyrate-producing bacteria, *Desulfovibrio* and *Enterococcus* faecalis in the feces of patients with colorectal cancer. *J. Gastroenterol. Hepatol.* 23 1298–1303. 10.1111/j.1440-1746.2008.05490.x 18624900

[B3] Baldwin R. L. Wu S. Li W. Li C. Bequette B. J. Li R. W. (2012). Quantification of transcriptome responses of the rumen epithelium to butyrate infusion using RNA-seq technology. *Gene Regul. Syst. Biol.* 6 67–80. 10.4137/GRSB.S9687 22654504 PMC3362330

[B4] Beauchemin K. A. Yang W. Z. Rode L. M. (2003). Effects of particle size of alfalfa-based dairy cow diets on chewing activity, ruminal fermentation, and milk production. *J. Dairy Sci.* 86 630–643. 10.3168/jds.s0022-0302(03)73641-8 12647969

[B5] Bi Y. Zeng S. Zhang R. Diao Q. Tu Y. (2018). Effects of dietary energy levels on rumen bacterial community composition in Holstein heifers under the same forage to concentrate ratio condition. *BMC Microbiol.* 18:69. 10.1186/s12866-018-1213-1219 29996759 PMC6042446

[B6] Blaser H. Dostert C. Mak T. W. Brenner D. (2016). TNF and ROS crosstalk in inflammation. *Trends Cell Biol.* 26 249–261. 10.1016/j.tcb.2015.12.002 26791157

[B7] Bolger A. M. Lohse M. Usadel B. (2014). Trimmomatic: a flexible trimmer for Illumina sequence data. *Bioinformatics* 30 2114–2120. 10.1093/bioinformatics/btu170 24695404 PMC4103590

[B8] Busby M. A. Stewart C. Miller C. A. Grzeda K. R. Marth G. T. (2013). Scotty: a web tool for designing RNA-Seq experiments to measure differential gene expression. *Bioinformatics* 29 656–657. 10.1093/bioinformatics/btt015 23314327 PMC3582267

[B9] Caporaso J. G. Bittinger K. Bushman F. D. DeSantis T. Z. Andersen G. L. Knight R. (2010). PyNAST: a flexible tool for aligning sequences to a template alignment. *Bioinformatics* 26 266–267. 10.1093/bioinformatics/btp636 19914921 PMC2804299

[B10] Chen G. J. Song S. D. Wang B. X. Zhang Z. F. Peng Z. L. Guo C. H. (2015). Effects of forage:concentrate ratio on growth performance, ruminal fermentation and blood metabolites in housing-feeding yaks. *Asian Austr. J Anim Sci.* 28 1736–1741. 10.5713/ajas.15.0419 26580441 PMC4647082

[B11] Chen Y. Chen Y. Shi C. Huang Z. Zhang Y. Li S. (2017). SOAPnuke: a MapReduce acceleration-supported software for integrated quality control and preprocessing of high-throughput sequencing data. *Gigascience* 7 1–6. 10.1093/gigascience/gix120 29220494 PMC5788068

[B12] Cohen J. (1988). *Statistical Power Analysis for the Behavioral Sciences*, 2nd Edn Hillsdale, NJ: Lawrence Erlbaum.

[B13] Dias J. Marcondes M. I. Noronha M. F. Resende R. T. Machado F. S. Mantovani H. C. (2017). Effect of pre-weaning diet on the ruminal archaeal, bacterial, and fungal communities of dairy calves. *Front Microbiol.* 8:1553. 10.3389/fmicb.2017.01553 28861065 PMC5559706

[B14] Dong Q. M. Zhao X. Q. Ma Y. S. Xu S. X. Li Q. Y. (2006). Live-weight gain, apparent digestibility, and economic benefits of yaks fed different diets during winter on the Tibetan plateau. *Livest Sci.* 101 199–207. 10.1016/j.livprodsci.2005.11.009

[B15] Edgar R. C. (2010). Search and clustering orders of magnitude faster than BLAST. *Bioinformatics* 26 2460–2461. 10.1093/bioinformatics/btq461 20709691

[B16] Edgar R. C. (2013). UPARSE: highly accurate OTU sequences from microbial amplicon reads. *Nat. Methods* 10 996–998. 10.1038/nmeth.2604 23955772

[B17] Edgar R. C. (2016). SINTAX: a simple non-Bayesian taxonomy classifier for 16S and ITS sequences. *bioRxiv* [preprint]. 10.1101/074161

[B18] Edgar R. C. Flyvbjerg H. (2015). Error filtering, pair assembly and error correction for next-generation sequencing reads. *Bioinformatics* 31 3476–3482. 10.1093/bioinformatics/btv401 26139637

[B19] Granja-Salcedo Y. T. Duarte Messana J. Carneiro de Souza V. Lino Dias A. V. Takeshi Kishi L. Rocha Rebelo L. (2017). Effects of partial replacement of maize in the diet with crude glycerin and/or soyabean oil on ruminal fermentation and microbial population in Nellore steers. *Br. J. Nutr.* 118 651–660. 10.1017/S0007114517002689 29185932

[B20] Guilloteau P. Martin L. Eeckhaut V. Ducatelle R. Zabielski R. Van Immerseel F. (2010). From the gut to the peripheral tissues: the multiple effects of butyrate. *Nutr Res Rev.* 23 366–384. 10.1017/S0954422410000247 20937167

[B21] Hosseini S. M. Ghorbani G. R. Rezamand P. Khorvash M. (2016). Determining optimum age of holstein dairy calves when adding chopped alfalfa hay to meal starter diets based on measures of growth and performance. *Animal* 10 607–615. 10.1017/S1751731115002499 26567925

[B22] Huang J. Li Y. (2018). Rumen methanogen and protozoal communities of tibetan sheep and gansu alpine finewool sheep grazing on the qinghai–tibetan plateau, China. *BMC Microbiol.* 18:212. 10.1186/s12866-018-1351-1350 30545295 PMC6293568

[B23] Ishii S. Kosaka T. Hori K. Hotta Y. Watanabe K. (2005). Coaggregation facilitates interspecies hydrogen transfer between *Pelotomaculum thermopropionicum* and *Methanothermobacter thermautotrophicus*. *Appl. Env. Microbiol.* 71 7838–7845. 10.1128/AEM.71.12.7838-7845.2005 16332758 PMC1317437

[B24] Jahani-Moghadam M. Mahjoubi E. Hossein Yazdi M. Cardoso F. C. Drackley J. K. (2015). Effects of alfalfa hay and its physical form (chopped versus pelleted) on performance of Holstein calves. *J. Dairy Sci.* 98 4055–4061. 10.3168/jds.2014-9126 25841969

[B25] Jiao J. Z. Li X. P. Beauchemin K. A. Tan Z. L. Tang S. X. Zhou C. S. (2015). Rumen development process in goats as affected by supplemental feeding *v. grazing*: age-related anatomic development, functional achievement and microbial colonisation. *Br. J. Nutr.* 113 888–900. 10.1017/S0007114514004413 25716279

[B26] Kargar S. Kanani M. (2019). Reconstituted versus dry alfalfa hay in starter feed diets of holstein dairy calves: effects on growth performance, nutrient digestibility, and metabolic indications of rumen development. *J. Dairy Sci.* 102 4051–4060. 10.3168/jds.2018-15153 30879820

[B27] Khafipour E. Li S. Plaizier J. C. Krause D. O. (2009). Rumen microbiome composition determined using two nutritional models of subacute ruminal acidosis. *Appl. Environ. Microbiol.* 75 7115–7124. 10.1128/AEM.00739-739 19783747 PMC2786511

[B28] Khan M. A. Bach A. Weary D. M. Von Keyserlingk M. A. G. (2016). Invited review: transitioning from milk to solid feed in dairy heifers. *J. Dairy Sci.* 99 885–902. 10.3168/jds.2015-9975 26709160

[B29] Khan M. A. Lee H. J. Lee W. S. Kim H. S. Kim S. B. Ki K. S. (2007). Starch source evaluation in calf starter: I. Feed consumption, body weight gain, structural growth, and blood metabolites in Holstein calves. *J Dairy Sci.* 90 5259–5268. 10.3168/jds.2007-0338 17954766

[B30] Khan M. A. Lee H. J. Lee W. S. Kim H. S. Kim S. B. Park S. B. (2008). Starch source evaluation in calf starter: II. Ruminal parameters, rumen development, nutrient digestibilities, and nitrogen utilization in Holstein calves. *J Dairy Sci.* 91 1140–1149. 10.3168/jds.2007-0337 18292270

[B31] Kim D. Langmead B. Salzberg S. L. (2015). HISAT: a fast spliced aligner with low memory requirements. *Nat. Methods* 12 357–360. 10.1038/nmeth.3317 25751142 PMC4655817

[B32] Kim Y. H. Nagata R. Ohtani N. Ichijo T. Ikuta K. Sato S. (2016). Effects of dietary forage and calf starter diet on ruminal pH and bacteria in Holstein calves during weaning transition. *Front Microbiol.* 7:1575. 10.3389/fmicb.2016.01575 27818645 PMC5073099

[B33] Kopecný J. Zorec M. Mrázek J. Kobayashi Y. Marinsek-Logar R. (2003). Butyrivibrio hungatei sp. nov. and *Pseudobutyrivibrio xylanivorans* sp. nov., butyrate-producing bacteria from the rumen. *Int. J. Syst. Evol. Microbiol.* 53 201–209. 10.1099/ijs.0.02345-0 12656174

[B34] Kosiorowska A. Puggaard L. Hedemann M. S. Sehested J. Jensen S. K. Kristensen N. B. (2011). Gastrointestinal development of dairy calves fed low-or high-starch concentrate at two milk allowances. *Animal* 5 211–219. 10.1017/S1751731110001710 22440766

[B35] Kverka M. Zakostelska Z. Klimesova K. Sokol D. Hudcovic T. Hrncir T. (2011). Oral administration of *Parabacteroides distasonis* antigens attenuates experimental murine colitis through modulation of immunity and microbiota composition. *Clin. Exp. Immunol.* 163 250–259. 10.1111/j.1365-2249.2010.04286.x 21087444 PMC3043316

[B36] Laarman A. H. Ruiz-Sanchez A. L. Sugino T. Guan L. L. Oba M. (2012). Effects of feeding a calf starter on molecular adaptations in the ruminal epithelium and liver of Holstein dairy calves. *J. Dairy Sci.* 95 2585–2594. 10.3168/jds.2011-4788 22541487

[B37] Langmead B. Salzberg S. L. (2012). Fast gapped-read alignment with Bowtie 2. *Nat. Methods* 9 357–359. 10.1038/nmeth.1923 22388286 PMC3322381

[B38] Leonard W. J. Lin J. X. (2000). Cytokine receptor signaling pathways. *J. Allergy Clin. Immunol.* 105 877–888. 10808165 10.1067/mai.2000.106899

[B39] Li B. Dewey C. N. (2011). RSEM: accurate transcript quantification from RNA-Seq data with or without a reference genome. *BMC Bioinformatics* 12:323. 10.1186/1471-2105-12-323 21816040 PMC3163565

[B40] Liu J. Bian G. Sun D. Zhu W. Mao S. (2017). Starter feeding supplementation alters colonic mucosal bacterial communities and modulates mucosal immune homeostasis in newborn lambs. *Front Microbiol.* 8:429. 10.3389/fmicb.2017.00429 28382025 PMC5361653

[B41] Livak K. J. Schmittgen T. D. (2001). Analysis of relative gene expression data using real-time quantitative PCR and the 2(-Delta Delta C(T)) Method. *Methods.* 25 402–408. 10.1006/meth.2001.1262 11846609

[B42] Loh G. Blaut M. (2012). Role of commensal gut bacteria in inflammatory bowel diseases. *Gut Microbes* 3 544–555. 10.4161/gmic.22156 23060017 PMC3495792

[B43] Long R. J. Ding L. M. Shang Z. H. Guo X. H. (2008). The yak grazing system on the Qinghai-Tibetan plateau and its status. *Rangel J.* 30 241–246.

[B44] Lozupone C. Knight R. (2005). UniFrac: a new phylogenetic method for comparing microbial communities. *Appl. Environ. Microbiol.* 71 8228–8235. 10.1128/aem.71.12.8228-8235.2005 16332807 PMC1317376

[B45] Lozupone C. A. Stombaugh J. Gonzalez A. Ackermann G. Wendel D. Vázquez-Baeza Y. (2013). Meta-analyses of studies of the human microbiota. *Genome Res.* 23 1704–1714. 10.1101/gr.151803.112 23861384 PMC3787266

[B46] Maggioli M. F. Palmer M. V. Thacker T. C. Vordermeier H. M. McGill J. L. Whelan A. O. (2016). Increased TNF-α/IFN-γ/IL-2 and decreased TNF-α/IFN-γ production by central memory T cells are associated with protective responses against bovine tuberculosis following BCG vaccination. *Front. Immunol.* 7:421. 10.3389/fimmu.2016.00421 27799930 PMC5066095

[B47] Michel T. A. Macy J. M. (1990). Generation of a membrane potential by sodium-dependent succinate efflux in Selenomonas ruminantium. *J. Bacteriol.* 172 1430–1435. 10.1128/jb.172.3.1430-1435.1990 2307654 PMC208616

[B48] Mirzaei M. Khorvash M. Ghorbani G. R. Kazemi-Bonchenari M. Riasi A. Nabipour A. (2015). Effects of supplementation level and particle size of alfalfa hay on growth characteristics and rumen development in dairy calves. *J. Anim. Physiol. Anim. Nutr.* 99 553–564. 10.1111/jpn.12229 25039298

[B49] Mukhopadhya I. Hansen R. Nicholl C. E. Alhaidan Y. A. Thomson J. M. Berry S. H. (2011). A comprehensive evaluation of colonic mucosal isolates of *Sutterella wadsworthensis* from inflammatory bowel disease. *PLoS One* 6:e27076. 10.1371/journal.pone.0027076 22073125 PMC3205041

[B50] Müller N. Worm P. Schink B. Stams A. J. Plugge C. M. (2010). Syntrophic butyrate and propionate oxidation processes: from genomes to reaction mechanisms. *Environ. Microbiol. Rep.* 2 489–499. 10.1111/j.1758-2229.2010.00147.x 23766220

[B51] Norouzian M. A. Valizadeh R. Vahmani P. (2011). Rumen development and growth of Balouchi lambs offered alfalfa hay pre-and post-weaning. *Trop. Anim. Health Proc.* 43 1169–1174. 10.1007/s11250-011-9819-z 21465104

[B52] O’Mahony L. McCarthy J. Kelly P. Hurley G. Luo F. Chen K. (2005). Lactobacillus and bifidobacterium in irritable bowel syndrome: symptom responses and relationship to cytokine profiles. *Gastroenterology* 128 541–551. 10.1053/j.gastro.2004.11.050 15765388

[B53] Orskov E. R. Benzie D. (1969). Studies on the oesophageal groove reflex in sheep and on the potential use of the groove to prevent the fermentation of food in the rumen. *Br. J. Nutr.* 23 415–420. 10.1079/bjn19690048 5787664

[B54] Paulson J. N. Stine O. C. Bravo H. C. Pop M. (2013). Differential abundance analysis for microbial marker-gene surveys. *Nat. Methods* 10 1200–1202. 10.1038/nmeth.2658 24076764 PMC4010126

[B55] Rey M. Enjalbert F. Combes S. Cauquil L. Bouchez O. Monteils V. (2014). Establishment of ruminal bacterial community in dairy calves from birth to weaning is sequential. *J. Appl. Microbiol.* 116 245–257. 10.1111/jam.12405 24279326

[B56] Romagnani S. (1992). Human TH1 and TH2 subsets: regulation of differentiation and role in protection and immunopathology. *Int. Arch. Allergy Immunol.* 98 279–285. 10.1159/000236199 1422257

[B57] Saro C. Hohenester U. M. Bernard M. Lagrée M. Martin C. Doreau M. (2018). Effectiveness of interventions to modulate the rumen microbiota composition and function in pre-ruminant and ruminant Lambs. *Front. Microbiol.* 9:1273. 10.3389/fmicb.2018.01273 29967596 PMC6015893

[B58] Sundberg C. Al-Soud W. A. Larsson M. Alm E. Yekta S. S. Svensson B. H. (2013). 454 pyrosequencing analyses of bacterial and archaeal richness in 21 full-scale biogas digesters. *FEMS Microbiol. Ecol.* 85 612–626. 10.1111/1574-6941.12148 23678985

[B59] Wang Q. Venkataramanan K. P. Huang H. Papoutsakis E. T. Wu C. H. (2013). Transcription factors and genetic circuits orchestrating the complex, multilayered response of *Clostridium acetobutylicum* to butanol and butyrate stress. *BMC Syst. Biol.* 7:120. 10.1186/1752-0509-7-120 24196194 PMC3828012

[B60] Wang W. Li C. Li F. Wang X. Zhang X. Liu T. (2016). Effects of early feeding on the host rumen transcriptome and bacterial diversity in lambs. *Sci. Rep.* 6:32479. 10.1038/srep32479 27576848 PMC5006043

[B61] Wang Y. Xu L. Liu J. Zhu W. Mao S. (2017). A high grain diet dynamically shifted the composition of mucosa-associated microbiota and induced mucosal injuries in the colon of sheep. *Front. Microbiol.* 8:2080. 10.3389/fmicb.2017.02080 29123511 PMC5662643

[B62] Weaver C. T. Harrington L. E. Mangan P. R. Gavrieli M. Murphy K. M. (2006). Th17: an effector CD4 T cell lineage with regulatory T cell ties. *Immunity* 24 677–688. 10.1016/j.immuni.2006.06.002 16782025

[B63] Wu S. Liu Y. Duan Y. Wang F. Guo F. Yan F. (2018). Intestinal toxicity of deoxynivalenol is limited by supplementation with Lactobacillus plantarum JM113 and consequentially altered gut microbiota in broiler chickens. *J. Anim. Sci. Biotechnol.* 9:74. 10.1186/s40104-018-0286-285 30338065 PMC6174567

[B64] Xie C. Mao X. Huang J. Ding Y. Wu J. Dong S. (2011). KOBAS 2.0: a web server for annotation and identification of enriched pathways and diseases. *Nucleic Acids Res.* 39 W316–W322. 10.1093/nar/gkr483 21715386 PMC3125809

[B65] Xie X. Yang C. Guan L. L. Wang J. Xue M. Liu J. X. (2018). Persistence of cellulolytic bacteria fibrobacter and treponema after short-term corn stover-based dietary intervention reveals the potential to improve rumen fibrolytic function. *Front. Microbiol.* 9:1363. 10.3389/fmicb.2018.01363 29997589 PMC6029512

[B66] Xue B. Zhao X. Q. Zhang Y. S. (2005). Seasonal changes in weight and body composition of yak grazing on alpine-meadow grassland in the Qinghai-Tibetan plateau of China. *J. Anim. Sci.* 83 1908–1913. 10.2527/2005.8381908x 16024711

[B67] Xue D. Chen H. Zhao X. Xu S. Hu L. Xu T. (2017). Rumen prokaryotic communities of ruminants under different feeding paradigms on the Qinghai-Tibetan Plateau. *Syst. Appl. Microbiol.* 40 227–236. 10.1016/j.syapm.2017.03.006 28495244

[B68] Yáñez-Ruiz D. R. Abecia L. Newbold C. J. (2015). Manipulating rumen microbiome and fermentation through interventions during early life: a review. *Front. Microbiol.* 6:1133. 10.3389/fmicb.2015.01133 26528276 PMC4604304

[B69] Yang B. He B. Wang S. S. Liu J. X. Wang J. K. (2015). Early supplementation of starter pellets with alfalfa improves the performance of pre-and postweaning Hu lambs. *J. Anim. Sci.* 93 4984–4994. 10.2527/jas.2015-9266 26523591

[B70] Yang B. Le J. Wu P. Liu J. Guan L. L. Wang J. (2018). Alfalfa intervention alters rumen microbial community development in hu lambs during early life. *Front. Microbiol.* 9:574. 10.3389/fmicb.2018.00574 29636743 PMC5881016

[B71] Yu Y. Lee C. Kim J. Hwang S. (2005). Group-specific primer and probe sets to detect methanogenic communities using quantitative real-time polymerase chain reaction. *Biotechnol. Bioeng.* 89 670–679. 10.1002/bit.20347 15696537

[B72] Zhang J. Shi H. Wang Y. Li S. Cao Z. Ji S. (2017). Effect of dietary forage to concentrate ratios on dynamic profile changes and interactions of ruminal microbiota and metabolites in holstein heifers. *Front. Microbiol.* 8:2206. 10.3389/fmicb.2017.02206 29170660 PMC5684179

[B73] Zi X. D. Zhong G. H. Wen Y. L. Zhong J. C. Liu C. L. Ni Y. A. (2004). Growth performance, carcass composition and meat quality of jiulong-yak (*Bos grunniens*). *Asian Austr. J. Anim. Sci.* 17 410–414. 10.5713/ajas.2004.410

