# Supplementary material for: Effect of Alfalfa Hay and Starter Feeding Intervention on Gastrointestinal Microbial Community, Growth and Immune Performance of Yak Calves
Source: Front Microbiol. 2020 Jun 4;11:994. doi: 10.3389/fmicb.2020.00994 (PMC7287295; doi:10.3389/fmicb.2020.00994)
Supplement: FILE 1: FIGURE S1 — The numbers of identified up-regulated and down-regulated DEGs of differential compared groups (SA vs. S, SA vs. A, SA vs. CON, S vs. A, S vs. CON, and A vs. CON). [file Data_Sheet_1.docx]

**Title:** Effect of alfalfa hay and starter feeding intervention on gastrointestinal microbial community, growth and immune performance of yak calves

**Authors:** Zhanhong Cui^*, #, ¶^, Shengru Wu^*, ¶, 1^, Jilan Li^#^, Qien Yang^&^, Shatuo Chai^#^, Lei Wang^#^, Xun Wang^#^, Xiaowei Zhang^#^, Shujie Liu^#, 1^, Junhu Yao^*, 1^

**Institution:** * College of Animal Science and Technology, Northwest A&F University, Yangling, Shaanxi, China

# Qinghai Academy of Animal Husbandry and Veterinary Sciences, Qinghai University, Xining, Qinghai, China

^&^ Northwest Plateau Institute of Biology, Chinese Academy of Sciences, Xining, Qinghai, China

co-first author: these 2 authors contributed equally to this work

**^1^ Co-corresponding authors:**

Shengru Wu, email: wushengru2013@163.com;

Shujie Liu, email: [mkylshj@126.com](mailto:mkylshj@126.com) (S. J. Liu);

Junhu Yao, email: [yaojunhu2004@sohu.com](mailto:yaojunhu2004@sohu.com) (J. H. Yao); Tel.: +86 13891883031.

**Supporting information**

**Table S1. Statistical data and quality controlled data of the RNA-Sequencing reads for six samples.**

**Table S2. The significantly enriched pathways based on the identified DEGs of different compared groups includes SA VS CON, S VS CON, and A VS CON.**

**Figure S1. The numbers of identified up-regulated and down-regulated DEGs of differential compared groups includes SA VS S, SA VS A, SA VS CON, S VS A, S VS CON, and A VS CON.**

**Supplementary information**

**Table S1.** **Statistical data and quality controlled data of the RNA-Sequencing reads for all samples.**

| Sample ID | Total Raw Reads(Mb) | Total Clean Reads(Mb) | Total Clean Bases(Gb) | Clean Reads Q20(%) | Clean Reads Q30(%) | Clean Reads Ratio(%) |
| --- | --- | --- | --- | --- | --- | --- |
| CON-1 | 43.68 | 41.03 | 6.15 | 97.53 | 92.41 | 93.93 |
| CON-2 | 48.72 | 45.74 | 6.86 | 97.54 | 92.46 | 93.89 |
| CON-3 | 44.37 | 41.22 | 6.18 | 97.91 | 93.46 | 92.89 |
| CON-4 | 44.46 | 41.83 | 6.27 | 97.74 | 92.93 | 94.09 |
| CON-5 | 44.79 | 41.79 | 6.27 | 97.31 | 91.85 | 93.30 |
| A-1 | 44.06 | 41.34 | 6.20 | 97.47 | 92.25 | 93.81 |
| A-2 | 49.83 | 46.87 | 7.03 | 97.51 | 92.35 | 94.06 |
| A-3 | 40.55 | 37.99 | 5.70 | 97.38 | 92.02 | 93.69 |
| A-4 | 48.31 | 45.20 | 6.78 | 97.44 | 92.16 | 93.57 |
| A-5 | 46.33 | 43.24 | 6.49 | 97.30 | 91.85 | 93.33 |
| S-1 | 49.25 | 45.53 | 6.83 | 97.80 | 93.16 | 92.45 |
| S-2 | 50.34 | 46.65 | 7.00 | 97.88 | 93.36 | 92.66 |
| S-3 | 47.97 | 44.43 | 6.66 | 97.75 | 93.02 | 92.63 |
| S-4 | 43.19 | 39.98 | 6.00 | 97.71 | 92.91 | 92.57 |
| S-5 | 40.50 | 37.94 | 5.69 | 97.45 | 92.22 | 93.69 |
| SA-1 | 47.58 | 44.07 | 6.61 | 97.81 | 93.18 | 92.64 |
| SA-2 | 43.10 | 40.03 | 6.00 | 97.67 | 92.86 | 92.88 |
| SA-3 | 48.60 | 44.99 | 6.75 | 97.72 | 92.97 | 92.58 |
| SA-4 | 47.94 | 44.68 | 6.70 | 98.09 | 93.94 | 93.20 |
| SA-5 | 42.07 | 39.13 | 5.87 | 98.04 | 93.78 | 93.02 |

**Table S2. The significantly enriched pathways based on the identified DEGs of different compared groups includes SA VS CON, S VS CON, and A VS CON.**

| **Compared group** | **Pathway** | **DEGs genes** | **All genes** | **P value** |
| --- | --- | --- | --- | --- |
| **SA vs CON** | **Allograft rejection** | **12 (2.26%)** | **155 (0.46%)** | **0.00** |
|  | **Graft-versus-host disease** | **11 (2.07%)** | **132 (0.39%)** | **0.00** |
|  | **Autoimmune thyroid disease** | **12 (2.26%)** | **168 (0.5%)** | **0.00** |
|  | **Cell adhesion molecules (CAMs)** | **21 (3.95%)** | **461 (1.36%)** | **0.00** |
|  | **Staphylococcus aureus infection** | **13 (2.44%)** | **205 (0.61%)** | **0.00** |
|  | **Type I diabetes mellitus** | **11 (2.07%)** | **158 (0.47%)** | **0.00** |
|  | **Glutamatergic synapse** | **14 (2.63%)** | **281 (0.83%)** | **0.00** |
|  | **Asthma** | **9 (1.69%)** | **125 (0.37%)** | **0.00** |
|  | **Nucleotide excision repair** | **16 (3.01%)** | **352 (1.04%)** | **0.00** |
|  | **Aldosterone synthesis and secretion** | **13 (2.44%)** | **255 (0.75%)** | **0.00** |
|  | **Retrograde endocannabinoid signaling** | **13 (2.44%)** | **260 (0.77%)** | **0.00** |
|  | **Salivary secretion** | **14 (2.63%)** | **305 (0.9%)** | **0.00** |
|  | **Insulin secretion** | **11 (2.07%)** | **209 (0.62%)** | **0.00** |
|  | **Phosphatidylinositol signaling system** | **14 (2.63%)** | **319 (0.94%)** | **0.00** |
|  | **Taste transduction** | **14 (2.63%)** | **319 (0.94%)** | **0.00** |
|  | **Intestinal immune network for IgA production** | **9 (1.69%)** | **152 (0.45%)** | **0.00** |
|  | **Th1 and Th2 cell differentiation** | **13 (2.44%)** | **297 (0.88%)** | **0.00** |
|  | **Viral myocarditis** | **13 (2.44%)** | **298 (0.88%)** | **0.00** |
|  | **Malaria** | **9 (1.69%)** | **164 (0.49%)** | **0.00** |
|  | **Prion diseases** | **7 (1.32%)** | **107 (0.32%)** | **0.00** |
|  | **Thyroid hormone synthesis** | **9 (1.69%)** | **171 (0.51%)** | **0.00** |
|  | **Rheumatoid arthritis** | **11 (2.07%)** | **250 (0.74%)** | **0.00** |
|  | **Antigen processing and presentation** | **11 (2.07%)** | **250 (0.74%)** | **0.00** |
|  | **Circadian entrainment** | **12 (2.26%)** | **288 (0.85%)** | **0.00** |
|  | **ABC transporters** | **8 (1.5%)** | **153 (0.45%)** | **0.00** |
|  | **Nicotine addiction** | **6 (1.13%)** | **92 (0.27%)** | **0.00** |
|  | **Steroid hormone biosynthesis** | **8 (1.5%)** | **158 (0.47%)** | **0.00** |
|  | **Adrenergic signaling in cardiomyocytes** | **15 (2.82%)** | **445 (1.32%)** | **0.01** |
|  | **African trypanosomiasis** | **6 (1.13%)** | **100 (0.3%)** | **0.01** |
|  | **Chagas disease (American trypanosomiasis)** | **11 (2.07%)** | **280 (0.83%)** | **0.01** |
|  | **Phototransduction - fly** | **7 (1.32%)** | **138 (0.41%)** | **0.01** |
|  | **Calcium signaling pathway** | **18 (3.38%)** | **593 (1.75%)** | **0.01** |
|  | **Inflammatory bowel disease (IBD)** | **8 (1.5%)** | **175 (0.52%)** | **0.01** |
|  | **NF-kappa B signaling pathway** | **11 (2.07%)** | **297 (0.88%)** | **0.01** |
|  | **Melanogenesis** | **10 (1.88%)** | **261 (0.77%)** | **0.01** |
|  | **Serotonergic synapse** | **10 (1.88%)** | **262 (0.78%)** | **0.01** |
|  | **cAMP signaling pathway** | **17 (3.2%)** | **576 (1.7%)** | **0.01** |
|  | **Renin secretion** | **8 (1.5%)** | **192 (0.57%)** | **0.01** |
|  | **Breast cancer** | **13 (2.44%)** | **403 (1.19%)** | **0.01** |
|  | **Bile secretion** | **8 (1.5%)** | **196 (0.58%)** | **0.01** |
|  | **Aldosterone-regulated sodium reabsorption** | **6 (1.13%)** | **123 (0.36%)** | **0.01** |
|  | **Systemic lupus erythematosus** | **11 (2.07%)** | **321 (0.95%)** | **0.01** |
|  | **Pancreatic secretion** | **9 (1.69%)** | **240 (0.71%)** | **0.01** |
|  | **GABAergic synapse** | **8 (1.5%)** | **202 (0.6%)** | **0.02** |
|  | **Rap1 signaling pathway** | **19 (3.57%)** | **699 (2.07%)** | **0.02** |
|  | **Pathways in cancer** | **27 (5.08%)** | **1105 (3.27%)** | **0.02** |
|  | **Endocrine and other factor-regulated calcium reabsorption** | **6 (1.13%)** | **130 (0.38%)** | **0.02** |
|  | **Metabolism of xenobiotics by cytochrome P450** | **6 (1.13%)** | **132 (0.39%)** | **0.02** |
|  | **Inositol phosphate metabolism** | **8 (1.5%)** | **218 (0.65%)** | **0.02** |
|  | **Protein digestion and absorption** | **19 (3.57%)** | **732 (2.17%)** | **0.02** |
|  | **Thyroid hormone signaling pathway** | **11 (2.07%)** | **352 (1.04%)** | **0.02** |
|  | **Gastric acid secretion** | **8 (1.5%)** | **222 (0.66%)** | **0.03** |
|  | **Notch signaling pathway** | **7 (1.32%)** | **181 (0.54%)** | **0.03** |
|  | **Retinol metabolism** | **6 (1.13%)** | **145 (0.43%)** | **0.03** |
|  | **Pyruvate metabolism** | **5 (0.94%)** | **113 (0.33%)** | **0.03** |
|  | **Proximal tubule bicarbonate reclamation** | **3 (0.56%)** | **45 (0.13%)** | **0.03** |
|  | **Amphetamine addiction** | **7 (1.32%)** | **197 (0.58%)** | **0.04** |
|  | **Long-term potentiation** | **7 (1.32%)** | **200 (0.59%)** | **0.04** |
|  | **Phenylalanine metabolism** | **3 (0.56%)** | **49 (0.15%)** | **0.04** |
|  | **Glyoxylate and dicarboxylate metabolism** | **4 (0.75%)** | **83 (0.25%)** | **0.04** |
|  | **Cytokine-cytokine receptor interaction** | **13 (2.44%)** | **487 (1.44%)** | **0.05** |
|  | **Basal cell carcinoma** | **5 (0.94%)** | **124 (0.37%)** | **0.05** |
|  | **Measles** | **10 (1.88%)** | **344 (1.02%)** | **0.05** |
|  | **Inflammatory mediator regulation of TRP channels** | **9 (1.69%)** | **299 (0.88%)** | **0.05** |
| **S vs CON** | **Asthma** | **16 (7.02%)** | **125 (0.37%)** | **0.00** |
|  | **Allograft rejection** | **17 (7.46%)** | **155 (0.46%)** | **0.00** |
|  | **Autoimmune thyroid disease** | **17 (7.46%)** | **168 (0.5%)** | **0.00** |
|  | **Intestinal immune network for IgA production** | **16 (7.02%)** | **152 (0.45%)** | **0.00** |
|  | **Staphylococcus aureus infection** | **16 (7.02%)** | **205 (0.61%)** | **0.00** |
|  | **Rheumatoid arthritis** | **16 (7.02%)** | **250 (0.74%)** | **0.00** |
|  | **Viral myocarditis** | **17 (7.46%)** | **298 (0.88%)** | **0.00** |
|  | **Systemic lupus erythematosus** | **17 (7.46%)** | **321 (0.95%)** | **0.00** |
|  | **Graft-versus-host disease** | **12 (5.26%)** | **132 (0.39%)** | **0.00** |
|  | **Type I diabetes mellitus** | **12 (5.26%)** | **158 (0.47%)** | **0.00** |
|  | **Malaria** | **12 (5.26%)** | **164 (0.49%)** | **0.00** |
|  | **NF-kappa B signaling pathway** | **15 (6.58%)** | **297 (0.88%)** | **0.00** |
|  | **Measles** | **15 (6.58%)** | **344 (1.02%)** | **0.00** |
|  | **Cell adhesion molecules (CAMs)** | **17 (7.46%)** | **461 (1.36%)** | **0.00** |
|  | **Inflammatory bowel disease (IBD)** | **11 (4.82%)** | **175 (0.52%)** | **0.00** |
|  | **Th1 and Th2 cell differentiation** | **13 (5.7%)** | **297 (0.88%)** | **0.00** |
|  | **Calcium signaling pathway** | **18 (7.89%)** | **593 (1.75%)** | **0.00** |
|  | **Antigen processing and presentation** | **12 (5.26%)** | **250 (0.74%)** | **0.00** |
|  | **Transcriptional misregulation in cancer** | **16 (7.02%)** | **535 (1.58%)** | **0.00** |
|  | **Th17 cell differentiation** | **11 (4.82%)** | **302 (0.89%)** | **0.00** |
|  | **Chagas disease (American trypanosomiasis)** | **10 (4.39%)** | **280 (0.83%)** | **0.00** |
|  | **HTLV-I infection** | **16 (7.02%)** | **703 (2.08%)** | **0.00** |
|  | **T cell receptor signaling pathway** | **10 (4.39%)** | **315 (0.93%)** | **0.00** |
|  | **Metabolism of xenobiotics by cytochrome P450** | **6 (2.63%)** | **132 (0.39%)** | **0.00** |
|  | **Primary immunodeficiency** | **5 (2.19%)** | **91 (0.27%)** | **0.00** |
|  | **African trypanosomiasis** | **5 (2.19%)** | **100 (0.3%)** | **0.00** |
|  | **Hematopoietic cell lineage** | **7 (3.07%)** | **225 (0.67%)** | **0.00** |
|  | **Leishmaniasis** | **6 (2.63%)** | **177 (0.52%)** | **0.00** |
|  | **B cell receptor signaling pathway** | **6 (2.63%)** | **198 (0.59%)** | **0.00** |
|  | **Natural killer cell mediated cytotoxicity** | **7 (3.07%)** | **267 (0.79%)** | **0.00** |
|  | **Phagosome** | **9 (3.95%)** | **445 (1.32%)** | **0.00** |
|  | **Salivary secretion** | **7 (3.07%)** | **305 (0.9%)** | **0.00** |
|  | **Chemical carcinogenesis** | **5 (2.19%)** | **172 (0.51%)** | **0.01** |
|  | **Rap1 signaling pathway** | **11 (4.82%)** | **699 (2.07%)** | **0.01** |
|  | **Fc epsilon RI signaling pathway** | **5 (2.19%)** | **195 (0.58%)** | **0.01** |
|  | **Bile secretion** | **5 (2.19%)** | **196 (0.58%)** | **0.01** |
|  | **Ras signaling pathway** | **11 (4.82%)** | **747 (2.21%)** | **0.01** |
|  | **Retinol metabolism** | **4 (1.75%)** | **145 (0.43%)** | **0.02** |
|  | **ABC transporters** | **4 (1.75%)** | **153 (0.45%)** | **0.02** |
|  | **Steroid hormone biosynthesis** | **4 (1.75%)** | **158 (0.47%)** | **0.02** |
|  | **Nicotinate and nicotinamide metabolism** | **3 (1.32%)** | **95 (0.28%)** | **0.03** |
|  | **Dilated cardiomyopathy** | **6 (2.63%)** | **348 (1.03%)** | **0.03** |
|  | **Nucleotide excision repair** | **6 (2.63%)** | **352 (1.04%)** | **0.03** |
|  | **Fc gamma R-mediated phagocytosis** | **5 (2.19%)** | **277 (0.82%)** | **0.04** |
|  | **Drug metabolism - cytochrome P450** | **3 (1.32%)** | **115 (0.34%)** | **0.04** |
| **A vs CON** | **TNF signaling pathway** | **1 (0.31%)** | **259 (0.77%)** | **0.00** |
|  | **Autoimmune thyroid disease** | **31 (9.66%)** | **168 (0.5%)** | **0.00** |
|  | **Pertussis** | **2 (0.62%)** | **223 (0.66%)** | **0.00** |
|  | **Regulation of lipolysis in adipocytes** | **2 (0.62%)** | **127 (0.38%)** | **0.00** |
|  | **Cell cycle** | **1 (0.31%)** | **302 (0.89%)** | **0.00** |
|  | **Nicotine addiction** | **6 (1.87%)** | **92 (0.27%)** | **0.00** |
|  | **Oxytocin signaling pathway** | **3 (0.93%)** | **430 (1.27%)** | **0.00** |
|  | **Malaria** | **20 (6.23%)** | **164 (0.49%)** | **0.00** |
|  | **Pancreatic secretion** | **2 (0.62%)** | **240 (0.71%)** | **0.00** |
|  | **Longevity regulating pathway - multiple species** | **2 (0.62%)** | **140 (0.41%)** | **0.00** |
|  | **Type I diabetes mellitus** | **20 (6.23%)** | **158 (0.47%)** | **0.00** |
|  | **Progesterone-mediated oocyte maturation** | **1 (0.31%)** | **248 (0.73%)** | **0.00** |
|  | **Phototransduction** | **1 (0.31%)** | **97 (0.29%)** | **0.00** |
|  | **Central carbon metabolism in cancer** | **1 (0.31%)** | **139 (0.41%)** | **0.00** |
|  | **Transcriptional misregulation in cancer** | **31 (9.66%)** | **535 (1.58%)** | **0.00** |
|  | **Parkinson's disease** | **1 (0.31%)** | **358 (1.06%)** | **0.00** |
|  | **Retrograde endocannabinoid signaling** | **7 (2.18%)** | **260 (0.77%)** | **0.00** |
|  | **Prolactin signaling pathway** | **2 (0.62%)** | **204 (0.6%)** | **0.00** |
|  | **Cytokine-cytokine receptor interaction** | **6 (1.87%)** | **487 (1.44%)** | **0.00** |
|  | **MicroRNAs in cancer** | **6 (1.87%)** | **486 (1.44%)** | **0.00** |
|  | **Oxidative phosphorylation** | **1 (0.31%)** | **330 (0.98%)** | **0.00** |
|  | **Complement and coagulation cascades** | **6 (1.87%)** | **261 (0.77%)** | **0.01** |
|  | **Breast cancer** | **9 (2.8%)** | **403 (1.19%)** | **0.01** |
|  | **Acute myeloid leukemia** | **2 (0.62%)** | **146 (0.43%)** | **0.01** |
|  | **Vasopressin-regulated water reabsorption** | **1 (0.31%)** | **121 (0.36%)** | **0.01** |
|  | **Endocytosis** | **6 (1.87%)** | **864 (2.56%)** | **0.01** |
|  | **AMPK signaling pathway** | **1 (0.31%)** | **307 (0.91%)** | **0.01** |
|  | **Mineral absorption** | **1 (0.31%)** | **161 (0.48%)** | **0.01** |
|  | **Aldosterone synthesis and secretion** | **1 (0.31%)** | **255 (0.75%)** | **0.01** |
|  | **Peroxisome** | **1 (0.31%)** | **241 (0.71%)** | **0.01** |
|  | **Morphine addiction** | **3 (0.93%)** | **223 (0.66%)** | **0.01** |
|  | **Hepatitis B** | **4 (1.25%)** | **324 (0.96%)** | **0.01** |
|  | **Legionellosis** | **2 (0.62%)** | **147 (0.44%)** | **0.01** |
|  | **Amoebiasis** | **10 (3.12%)** | **744 (2.2%)** | **0.01** |
|  | **Type II diabetes mellitus** | **2 (0.62%)** | **133 (0.39%)** | **0.02** |
|  | **Lysosome** | **3 (0.93%)** | **392 (1.16%)** | **0.02** |
|  | **Dilated cardiomyopathy** | **15 (4.67%)** | **348 (1.03%)** | **0.02** |
|  | **Leishmaniasis** | **10 (3.12%)** | **177 (0.52%)** | **0.02** |
|  | **Basal transcription factors** | **1 (0.31%)** | **137 (0.41%)** | **0.02** |
|  | **Pyruvate metabolism** | **2 (0.62%)** | **113 (0.33%)** | **0.02** |
|  | **Tuberculosis** | **11 (3.43%)** | **508 (1.5%)** | **0.02** |
|  | **Amyotrophic lateral sclerosis (ALS)** | **2 (0.62%)** | **172 (0.51%)** | **0.02** |
|  | **Toxoplasmosis** | **3 (0.93%)** | **295 (0.87%)** | **0.03** |
|  | **NOD-like receptor signaling pathway** | **2 (0.62%)** | **436 (1.29%)** | **0.03** |
|  | **Ribosome biogenesis in eukaryotes** | **1 (0.31%)** | **211 (0.62%)** | **0.03** |
|  | **Biosynthesis of unsaturated fatty acids** | **2 (0.62%)** | **59 (0.17%)** | **0.03** |
|  | **African trypanosomiasis** | **10 (3.12%)** | **100 (0.3%)** | **0.03** |
|  | **Chemical carcinogenesis** | **6 (1.87%)** | **172 (0.51%)** | **0.04** |
|  | **Thyroid hormone synthesis** | **1 (0.31%)** | **171 (0.51%)** | **0.04** |
|  | **Mucin type O-glycan biosynthesis** | **1 (0.31%)** | **58 (0.17%)** | **0.04** |
|  | **Metabolic pathways** | **31 (9.66%)** | **3429 (10.15%)** | **0.04** |
|  | **mTOR signaling pathway** | **3 (0.93%)** | **386 (1.14%)** | **0.05** |
|  | **Allograft rejection** | **30 (9.35%)** | **155 (0.46%)** | **0.05** |
|  | **Proximal tubule bicarbonate reclamation** | **1 (0.31%)** | **45 (0.13%)** | **0.05** |


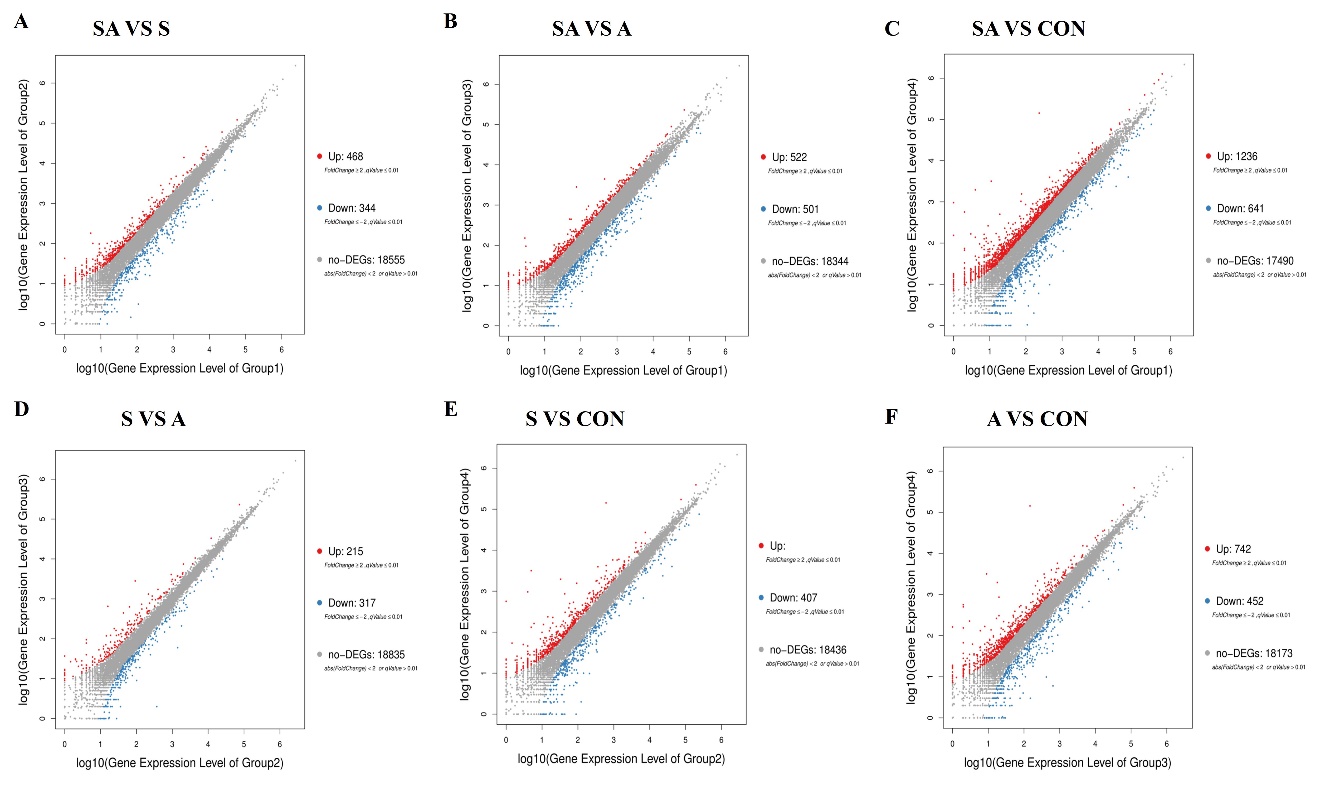


**Figure S1. The numbers of identified up-regulated and down-regulated DEGs of differential compared groups includes SA VS S, SA VS A, SA VS CON, S VS A, S VS CON, and A VS CON.**
